# A Core Human Microbiome as Viewed through 16S rRNA Sequence Clusters

**DOI:** 10.1371/journal.pone.0034242

**Published:** 2012-06-13

**Authors:** Susan M. Huse, Yuzhen Ye, Yanjiao Zhou, Anthony A. Fodor

**Affiliations:** 1 Josephine Bay Paul Center, Marine Biological Laboratory, Woods Hole, Massachusetts, United States of America; 2 School of Informatics and Computing, Indiana University, Bloomington, Indiana, United States of America; 3 The Genome Center, Washington University School of Medicine, Saint Louis, Missouri, United States of America; 4 Department of Bioinformatics and Genomics, University of North Carolina, Charlotte, North Carolina, United States of America; University of Hyderabad, India

## Abstract

We explore the microbiota of 18 body sites in over 200 individuals using sequences amplified V1–V3 and the V3–V5 small subunit ribosomal RNA (16S) hypervariable regions as part of the NIH Common Fund Human Microbiome Project. The body sites with the greatest number of core OTUs, defined as OTUs shared amongst 95% or more of the individuals, were the oral sites (saliva, tongue, cheek, gums, and throat) followed by the nose, stool, and skin, while the vaginal sites had the fewest number of OTUs shared across subjects. We found that commonalities between samples based on taxonomy could sometimes belie variability at the sub-genus OTU level. This was particularly apparent in the mouth where a given genus can be present in many different oral sites, but the sub-genus OTUs show very distinct site selection, and in the vaginal sites, which are consistently dominated by the *Lactobacillus* genus but have distinctly different sub-genus V1–V3 OTU populations across subjects. Different body sites show approximately a ten-fold difference in estimated microbial richness, with stool samples having the highest estimated richness, followed by the mouth, throat and gums, then by the skin, nasal and vaginal sites. Richness as measured by the V1–V3 primers was consistently higher than richness measured by V3–V5. We also show that when such a large cohort is analyzed at the genus level, most subjects fit the stool “enterotype” profile, but other subjects are intermediate, blurring the distinction between the enterotypes. When analyzed at the finer-scale, OTU level, there was little or no segregation into stool enterotypes, but in the vagina distinct biotypes were apparent. Finally, we note that even OTUs present in nearly every subject, or that dominate in some samples, showed orders of magnitude variation in relative abundance emphasizing the highly variable nature across individuals.

## Introduction

While the microbiota that live on and in the human body have long been recognized as critical to understanding a variety of human diseases, we are only beginning to understand their equally critical role in maintaining human health. To facilitate this understanding, the National Institutes of Health launched the Human Microbiome Project (HMP) in 2008 [1] to sequence the microbiome of healthy human subjects (http://commonfund.nih.gov/hmp). One of the primary goals of the HMP is to characterize the human microbiome of healthy individuals, and to describe, if possible, a core microbiome. The NIH enrolled over 200 healthy subjects, both male and female, and collected microbial DNA samples from 18 different body sites [2]. Researchers from many different academic institutions are part of the HMP Data Analysis Working Group analyzing the HMP sequence data to answer a number of fundamental questions for a basic understanding of a healthy human microbiome. The HMP is using both 16S rRNA tag sequencing to elucidate the types of microbes and their relative abundances and shotgun metagenomic sequencing to find out what functions these microbes may be performing. These analyses, being published as an overview manuscript [3] and a series of companion papers, lay the groundwork for further research in the human microbiome: the similarities and differences between individuals and body sites, and through time the numbers and types of microbes and what role they play in human health.

The 16S rRNA gene is considered the gold standard for phylogenetic studies of microbial communities and for assigning taxonomic names to bacteria. The explosion of sequence data brought about by Next-Generation Sequencing (NGS) is highlighting a richness of microbes not previously anticipated. NGS comes with a clear trade-off. The number of reads sequenced is greater by orders of magnitude than previous methods (e.g., Sanger sequencing), but the reads are much shorter. The read length using the Roche GS-FLX (‘454’) technology has been increasing rapidly from 100 nt in 2006 to over 400 nt at present. Unfortunately, taxonomists cannot provide taxonomic names for all of the novel organisms discovered by this unprecedented depth of sampling. Even in sections of the bacterial tree that are well described, existing tools are generally not sufficient to provide species names or phylogenetic information for the millions of short reads. For instance, the most commonly used tool for assigning taxonomy to 16S tags, the Ribosomal Database Project (RDP) Classifier [4], at best classifies 16S sequences only as far as the genus level, although many sequences that are distant from the commonly used reference sequences or that are taxonomically ambiguous can only be described to class, order or family levels. To complement analyses relying on limited taxonomic names, 16S rRNA sequences can be clustered together into operational taxonomic units (OTUs) at the 97% similarity (3% difference). This level of sequence-based clustering is generally recognized as providing differentiation of bacterial organisms below the genus level, although it would be inaccurate to assume that this level of clustering consistently defines either microbial species or strains.

Previous studies have demonstrated a great deal of variation in gut and nasal microbiota between individuals [5], [6], [7], [8], [9], and in the microbiota at different body sites within a single individual [10]. This study uses the largest number of healthy subjects to date to look for the possibility of a set of core OTUs common across individuals and body sites within the larger context of variation. Using the OTU approach, we analyze the HMP 16S tag-sequencing data to look for organisms that occur in most or all healthy subjects. The depth of sequencing per sample in this project is not adequate to understand the nature or extent of rare organisms that often play an important role in health and disease; instead, we focus on the more abundant organisms that are common across individuals.

## Results

### Study Set

The HMP 16S sequencing includes sequences amplified from the V3–V5 region, and in most cases amplified from the V1–V3 region as well. After filtering sequences using read quality and sample size requirements (see Methods), our data included 16S sequences from 238 subjects for the V3–V5 region and 168 subjects for the V1–V3 region. There were 112 female and 127 male subjects. Samples were collected from 18 body sites, including nine sites within the oral cavity: saliva, supragingival and subgingival plaque (plaque above and below the gum line), tongue dorsum (back of the tongue), hard palate (roof of the mouth), buccal mucosa (inside of the cheek), keratinized gingiva (gums), palatine tonsils (tonsils at the back of the mouth), and throat, four skin sites: left and right antecubital fossae (inner elbows), left and right retroauricular creases (behind the ears), three vaginal sites: mid-vagina, posterior fornix (the back of the vagina) and vaginal introitus (the entrance), one nasal site: the anterior nares (front of the nostrils), and one stool sample [2]. There were a total of ∼12 million V1–V3 and ∼15 million V3–V5 reads, and between 404 and 9,489 OTUs identified by body site, with an average sequencing depth of 5,709 tags (Table S1). OTUs were created with the bioinformatics package mothur using single-linkage preclustering and average linkage clustering (see Methods).

### Defining a Core Microbiome of Healthy Humans

The most stringent definition of a 16S OTU being a member of a core microbiome would require its presence within a body site in all subjects (100%) sampled. Using this definition, we found at least one core phylotype (here represented by a 3% OTU) seen in the V1–V3 sequences in each body site except the anterior nares, saliva and the three vaginal sites (Table 1, Table S2). For the V3–V5 sequences, an additional three body sites had no core OTUs (left and right antecubital fossae and subgingival plaque) while the anterior nares sample showed two core OTUs present in V3–V5 but none in V1–V3. These differences between V1–V3 and V3–V5 may reflect differences in the rate of evolution in different regions of the hypervariable regions of the 16S rRNA genes, as well as the ability of each region to differentiate microbial organisms.

**Table 1:** Number of Core OTUs present at different prevalence thresholds.

	100%		95%		90%		75%		50%	
Body Site	V1–V3	V3–V5	V1–V3	V3–V5	V1–V3	V3–V5	V1–V3	V3–V5	V1–V3	V3–V5
**Saliva**	0/0%	7/26%	12/28%	22/41%	23/39%	29/44%	44/47%	44/48%	79/52%	75/52%
**Supragingival plaque**	5/12%	4/19%	13/30%	15/38%	20/40%	22/45%	32/45%	32/50%	56/49%	57/57%
**Hard palate**	6/44%	8/47%	14/54%	16/58%	20/59%	21/61%	36/65%	31/63%	60/68%	51/68%
**Palatine Tonsils**	8/19%	3/16%	14/27%	16/42%	23/35%	21/46%	38/39%	32/49%	68/44%	61/57%
**Tongue dorsum**	6/26%	5/31%	15/44%	13/51%	21/49%	22/59%	33/57%	30/62%	49/60%	43/65%
**Throat**	4/16%	3/15%	15/32%	13/36%	22/37%	21/42%	34/41%	33/45%	57/45%	54/51%
**Buccal mucosa**	3/40%	6/50%	11/50%	11/59%	15/52%	17/63%	25/57%	24/64%	54/61%	47/68%
**Subgingival plaque**	1/4%	0/0%	10/20%	7/18%	16/23%	19/29%	36/35%	37/36%	67/40%	64/44%
**Keratinized gingiva**	2/36%	1/32%	3/39%	7/56%	6/43%	8/56%	14/53%	12/69%	33/64%	19/72%
**Anterior nares**	0/0%	2/17%	3/21%	4/32%	4/22%	4/32%	8/25%	6/34%	18/26%	18/37%
**Stool**	2/1%	1/3%	7/6%	5/8%	11/8%	7/9%	22/11%	20/11%	47/13%	50/15%
**Right Antecubital fossa**	1/9%	0/0%	1/9%	3/13%	2/11%	3/13%	7/15%	8/15%	16/17%	29/23%
**Left Antecubital fossa**	1/10%	0/0%	1/10%	2/8%	2/13%	3/10%	5/15%	10/12%	15/19%	34/21%
**Left Retroauricular crease**	1/24%	1/27%	2/35%	2/34%	3/37%	2/34%	5/40%	4/36%	8/41%	11/39%
**Right Retroauricular crease**	1/21%	1/23%	2/30%	2/29%	2/30%	3/31%	6/35%	4/31%	8/36%	8/33%
**Posterior fornix**	0/0%	0/0%	0/0%	1/38%	0/0%	1/38%	1/29%	1/38%	4/43%	2/39%
**Mid-vagina**	0/0%	0/0%	0/0%	1/41%	0/0%	1/41%	1/26%	1/41%	10/42%	4/42%
**Vaginal introitus**	0/0%	0/0%	0/0%	1/37%	0/0%	1/37%	2/28%	2/37%	12/43%	11/44%

Data represents the number of core OTUs found using either the V1-V3 or the V3-V5 regions of the 16S rRNA gene. Values are reported for OTUs present in 100%, 95%, 90%, 75%, and 50% of samples. The first number in each cell is the number of core OTUs for that body site, 16S region and prevalence. The second number is the percent of all sample tags for that body site and 16S region represented by the core OTUs.

As we will explore further, there is a wide variation in abundance across samples for even the most prevalent OTUs. Core OTUs that are dominant in some samples can be rare in others. Coupling this large variation in relative abundance with the fact that the sequence depth within nearly half of our samples was modest (fewer than 5,000 tags), we cannot assume that the lack of an OTU in any particular sample corresponds to its true absence in the subject – it may simply be below the detection level of a small sample. If we use a slightly less stringent threshold and define a core OTU as being present in 95% of all the samples of a body site, we see a more consistent view between primer sets with all body sites having at least one core OTU except the three vaginal samples using V1–V3. Using an even less stringent definition of 90% prevalence, we find even more OTUs as would be expected (Table S2), but the differences are minor. For our core OTU analyses therefore, we define “core OTUs” as those that are present in at least 95% of all samples for a given body site. Table S3 provides the OTU-level taxonomy for each of the 95% core OTUs.

**Figure 1:**
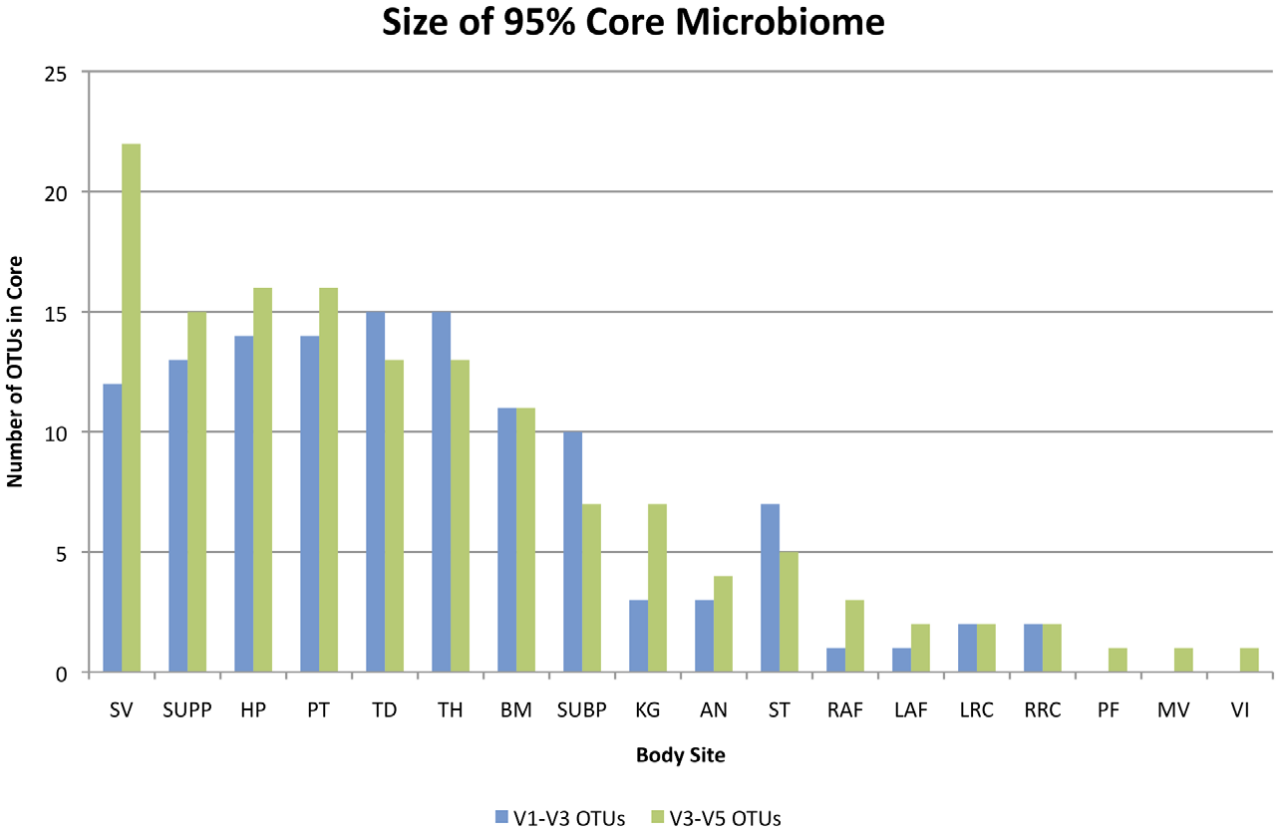
Size of the 95% core microbiome by body site. The oral cavity sites show the greatest number of core OTUs with both the V1–V3 and the V3–V5 tags, followed by the stool, the anterior nares, then the skin and the vaginal sites. Core OTUs are defined as those OTUs appearing in at least 95% of all samples for a given body site. Body site labels in order are: saliva (SV), supragingival plaque (SUPP), hard palate (HP), palatine tonsils (PT), tongue dorsum (TD), throat (TH), buccal mucosa (BM), subgingival plaque (SUBP), keratinized gingiva (KG), anterior nares (AN), stool (ST), right (RAF) and left (LAF) antecubital fossae, left (LRC) and right (RRC) retroauricular creases, posterior fornix (PF), mid-vagina (MV), and vaginal introitus (VI).

Examining the V3–V5 sequences for which we have more samples, we note that the oral cavity sites have a consistently richer core microbiome than other sites, ranging from 7 core V3–V5 OTUs in the keratinized gingiva and subgingival plaque to 22 in the saliva (Table 1, Figure 1), where we have defined core richness as a count of the number of OTUs found in 95% of the samples, rather than an estimate of the projected total richness of the bacterial community. The buccal mucosa, hard palate, palatine tonsils, supragingival plaque, throat, and tongue dorsum all had similar core richnesses of 11 to 16 V3–V5 OTUs. The V1–V3 OTUs showed similar patterns with the oral sites having a core V1–V3 richness of 10–15 OTUs except the keratinized gingiva with only 3 OTUs. The stool (5 V3–V5 and 7 V1–V3 OTUs), anterior nares (4 V3–V5 and 3 V1–V3 OTUs), and skin samples (2–3 V3–V5 and 1–2 V1–V3 OTUs) had similar size cores, and noticeably less than the oral cavity. The three vaginal sites had only one V3–V5 core OTU each but no cores by V1–V3. The *Lactobacillus* tags predominant in the vaginal sites were split into multiple V1–V3 OTUs, and no one *Lactobacillus* OTU was seen in even 90% of all vaginal samples, although 95% of the samples did have at least one of the three most common V1–V3 OTUs. In general, the core OTUs of a given body site represent less than 2% of the total number of different OTUs in that body site (results not shown).

**Figure 2:**
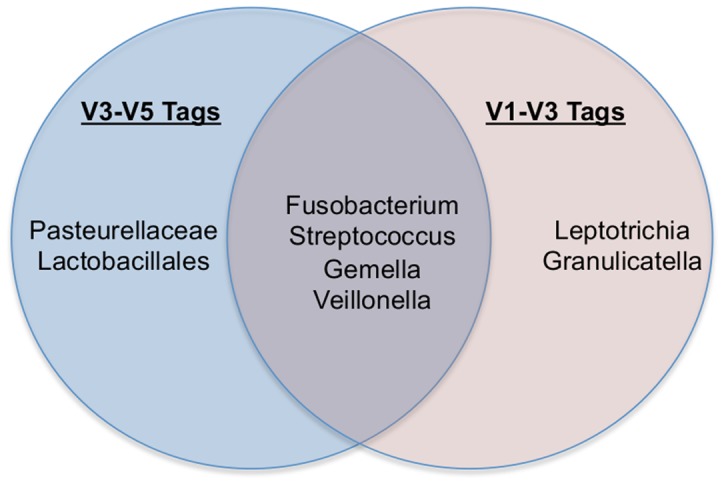
Core OTUs present in at least seven of the nine oral sites. While each 16S amplification (V1–V3 and V3–V5) has 6 core OTUs, there are differences between the amplifications, with Pasteurellaceae and Lactobacillales identified with the V3–V5 tags but not the V1–V3 tags, and *Leptotrichia* and *Granulicatella* (a member of the Lactobacillales order) identified with the V1–V3 tags but not the V3–V5 tags. *Fucobacterium*, *Gemella*, *Streptococcus*, and *Veillonella* were identified in both sets of OTUs.

Comparing across body site groups, there were 4 V3–V5 95% core OTUs present in all 9 oral sites representing *Fusobacterium*, *Streptococcus*, Pasteurellaceae, and *Veillonella*. *Granulicatella* and *Gemella* were present in samples from all oral sites, but in some cases with a prevalence of only 92–94%. Only 2 OTUs were present in all V1–V3 oral sites and both were *Streptococcus*, a third OTU representing *Veillonella* was present in all oral sites at a prevalence of 90% or more. Relaxing the requirement from all 9 to 7 oral sites picks up more core OTUs and shows more in common between the hypervariable regions (see Figure 2).

Across all skin samples, a *Propionibacterium* OTU was core at 99–100% in all four sites in both V1–V3 and V3–V5 data. A *Staphylococcus* V3–V5 OTU was core in three of the four skin sites, and nearly core at 93% prevalence in the fourth site. A V1–V3 *Staphylococcus* OTU was present in all 4 sites at greater 90% prevalence. No additional V1–V3 core OTUs were present in the skin samples. Only one V3–V5 OTU was core in the three vaginal sites, a *Lactobacillus*, and no OTUs were core with the V1–V3 data. The stool samples contained 7 core OTUs at a 95% prevalence representing several members of the Lachnospiraceae family as well as *Faecalibacterium*, *Oscillibacter* and two separate *Bacteroides* OTUs. The *Bacteroides* OTUs were by far the most abundant comprising on average 21% of the sequences.

We did not find a core microbiome across all subjects and body sites. The most common oral *Streptococcus* OTU in both the V1–V3 and the V3–V5 data was also the most prevalent OTU across all sites, being found also in the anterior nares, and as core in the two antecubital fossa sites with the V3–V5 sequencing. This lack of cross-body core OTUs is not surprising given that different body sites represent starkly different environments for adaption, include both internal and external sites, and have varying levels of moisture, acidity, and temperature, to name just a few differences.

### Core OTUs – Abundance and Prevalence

**Figure 3:**
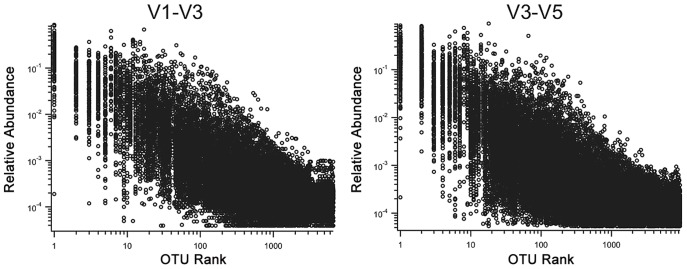
Relative abundance of OTUs. For each OTU, the relative abundance is plotted for each sample in which the OTU is present. The OTUs with the highest number of total sequences are ranked first and plotted at the leftmost side, and the OTUs with the lowest total number of sequences are ranked last and plotted toward the right. Figure 3A shows the V1–V3 OTUs and Figure 3B the V3–V5 OTUs. Even OTUs that are among the top 10 most abundant span at least 3 orders of magnitude of relative abundances from less than 0.01% to more than 10%.

Despite their prevalence across subjects, the relative abundances of core OTUs vary dramatically between subjects. To demonstrate the scope of individual variation in the composition and abundance of the microbial communities, we plotted the normalized counts of each V3–V5 OTU in each sample placing the most highly abundant OTUs (Figure 3). We see that even the most abundant core OTUs are highly variable across subjects with differences in relative abundance that span multiple orders of magnitude. For example, the most abundant OTU in the stool samples has a mean relative abundance of 0.23 (meaning that on average it accounts for 23% of all the sequences in each sample), but this relative abundance varies nearly 5,000-fold across our samples, ranging from 84% down to 0.021% (and not detected in seven samples). This same pattern in which OTUs display tremendous variation across different subjects was found repeatedly across the other body sites that were sampled (results not shown).

**Figure 4:**
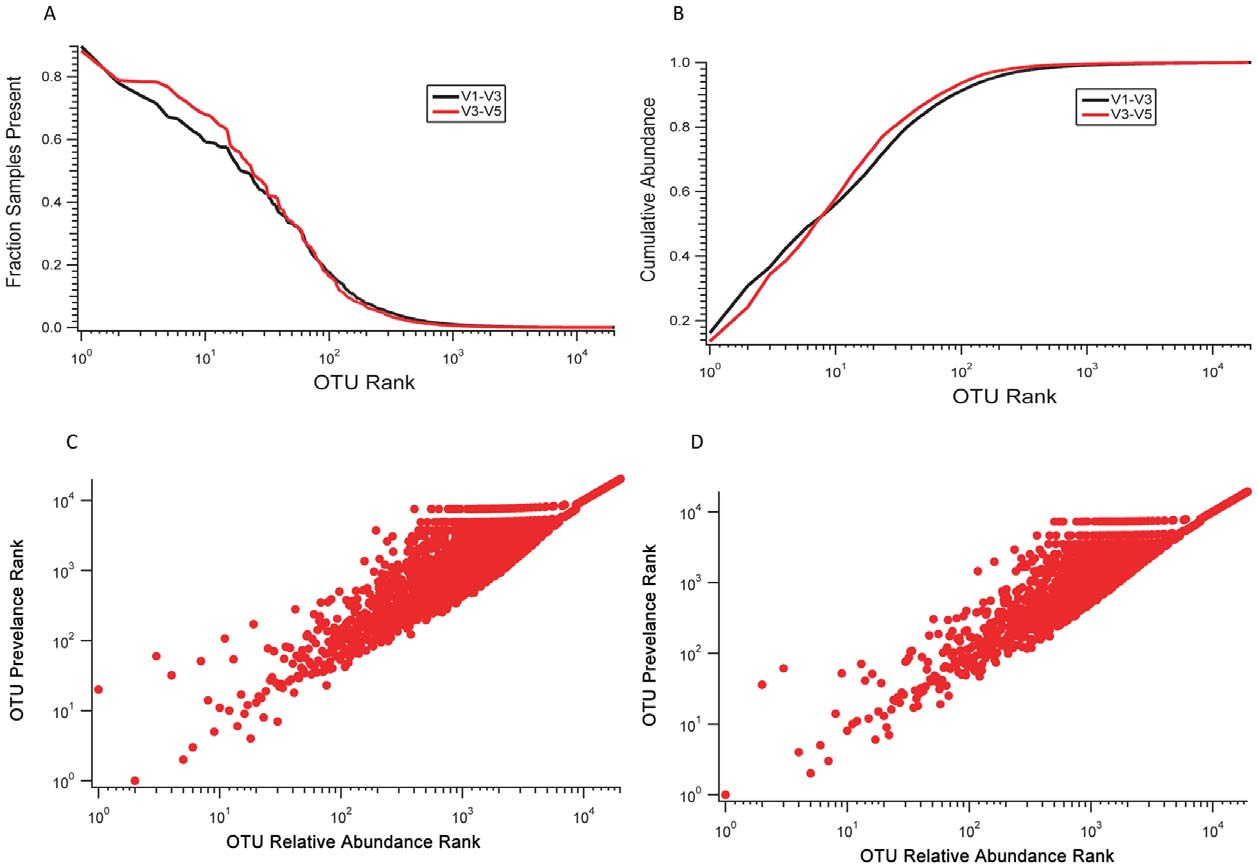
Comparison of OTU abundance and prevalence across all samples. The OTU rank is defined by the total number of sequences across all samples, with the most abundant ranked first (on the left). In panel A, OTU prevalence, the fraction of samples containing that OTU, is compared to OTU rank abundance. The most abundant OTUs appeared in a higher percentage of samples than the less abundant OTUs. In panel B, the cumulative abundance of OTUs as a function of OTU rank abundance showing that the 100 most abundant OTUs accounted for almost all of the sequence reads in both the V1–V3 and the V3–V5 amplifications. Panels C and D (V1–V3 and V3–V5 respectively) show the OTU prevalence rank against the OTU rank, with the most abundant OTUs tending also to be the most prevalent OTUs.

The prevalence of OTUs trends positively with their abundance (Figure 4). The most abundant OTUs tend to be present in more subjects than the less abundant OTUs overall. Obviously an OTU at any given size will have an increased overall abundance rank if it is present in more body sites and subjects. Presence in many subjects, however, will not substantially increase the rank abundance of an OTU that is only present in low numbers, and an OTU that is highly abundant (many thousand reads) will have a very low rank even if it is only present in a few subjects. While abundance and prevalence are not fully independent, they clearly correlate in the human microbiome. Figure 4 panel A shows the strong trend between the OTU rank based on overall abundance with the overall prevalence, as defined by the fraction of samples where the OTU is present. Panel B shows the OTU rank against the cumulative abundance of sequence tags, highlighting that the top 100 OTUs for both sets of tag data account for nearly all of the sequence tags. The lower panels compare the OTU rank against the prevalence rank for the V1–V3 (C) and the V3–V5 (D) tags. A general trend is apparent between the OTU abundance rank and prevalence rank, with the most abundant OTUs tending to be more prevalent as well. Highly prevalent but low abundance taxa were generally not detected by the level of sequencing effort provided by the HMP.

### Presence of Biome Types

Recent studies by Arumugam et al [8] and Wu et al [11] described between two and three biome types (enterotypes) consisting of clusters dominated by *Bacteroides, Ruminococcus* or Clostridiales, and *Prevotella*. To determine if clear enteric biome types were also present in the stool samples from the HMP cohort, we first used the RDP taxonomy directly (prior to using the OTUs) and assigned samples to draft biome types defined by their most abundant taxon. For the V3–V5 data, samples were assigned to *Bacteroides* (n = 192), *Prevotella* (n = 9), *Ruminococcus* (n = 2), *Alistipes* (n = 4), or *Oscillibacter* (n = 3). With the V1–V3 tags, we found slightly different types: *Bacteroides* (n = 100), *Prevotella* (n = 8), *Akkermansia* (n = 1), *Alistipes* (n = 1) and multiple Clostridiales (n = 10) including Ruminococcaceae (*Faecalibacterium*, *Hydrogenoanaerobacterium*, *Subdoligranulum*), Lachnospiraceae (*Coprococcus*, *Pseudobutyrivibrio*, *Catonella*) and Veillonellaceae (*Dialister*).

**Figure 5:**
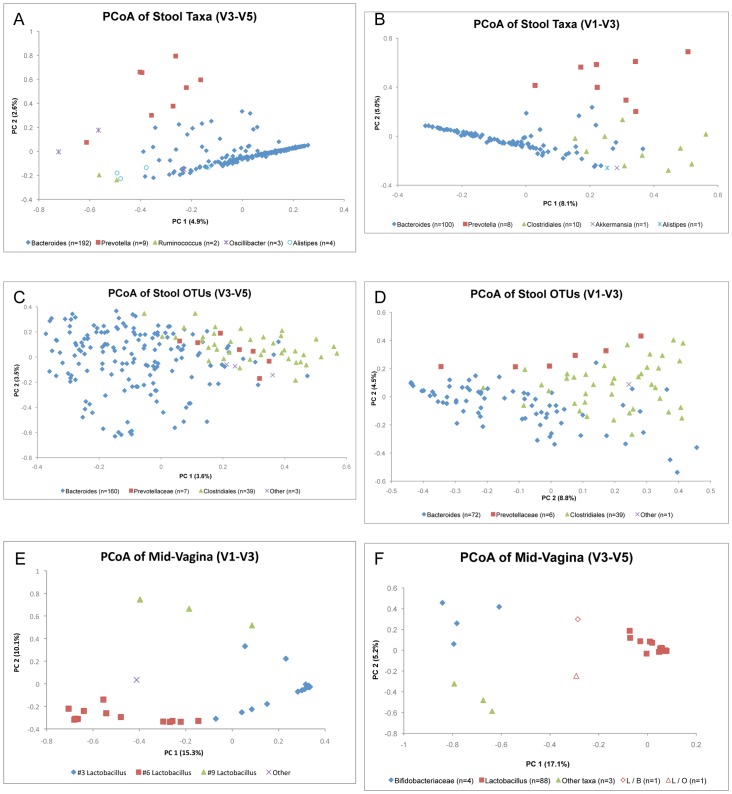
PCoA of Biome Types in stool and vaginal midpoint samples. Panels A and B are principle coordinates analyses of stool samples based on the RDP taxonomy and using Morisita-Horn distance. With the V3–V5 data, the *Bacteroides*-dominated subjects are segregated from both the *Ruminococcus*-dominated samples and the *Prevotella*-dominated samples. The *Alistipes* and *Oscillibacter* samples overlap with the other biome types. The *Bacteroides* and Clostridiales show greater overlap with the V1–V3 taxonomy (Panel B), while *Prevotella* is still segregated but not well separated. With both V3–V5 and V1–V3 data, the intra-biome type distances are as great as inter-biome type distances. At the OTU-level (Panels C and D) the *Bacteroides* and Clostridiales biome types have much greater overlap. The Prevotellaceae biome type has complete overlap with the other two biome types in the V3–V5 OTU data (Panel C) but mild segregation with V1–V3. At the OTU-level, the intra-biome type distances are greater than the inter-biome type distances. Panels E and F are the PCoA results for the mid-vagina samples, V3–V5 and V1–V3 respectively. The V3–V5 OTUs did not differentiate the *Lactobacillus* species, but show that while most subjects fall under the *Lactobacillus*-dominated type, there are also types dominated by either Bifidobacteriaceae or other taxa. The V1–V3 OTUs separated the subjects by *Lactobacillus* sub-types. Not all of the subjects classified as Bifidobacteriaceae with V3–V5 had corresponding samples large enough (>1000 tags) to be included in the V1–V3 plot and vice-versa.

A PCoA analysis based on the RDP taxonomy (Figure 5 A&B) shows that the samples assigned to *Bacteroides* and *Prevotella* can be segregated by the first two components, although these components together explain only a small amount of the community differences (8% for V3–V5 and 13% for V1–V3). The segregation is minimal, however, and two types do not form discrete, well-separated clusters. In the V3–V5 analysis, the *Alistipes* samples are located between the *Ruminococcus* and the *Bacteroides*, while the *Oscilllibacter* overlaps both the *Bacteroides* and the *Prevotella*. In the V1–V3 samples, the *Bacteroides* and Clostridiales have some separation but also a clear region of overlap shared by the single *Akkermansia* and *Alistipes* dominated samples. The data show community gradients rather than community clusters with a continuous ratio of Prevotella to Bacteroides. The differentiation between biome types occurs simply at the point when the ratio becomes greater than one, not when there is a large separation of types. This is highlighted by comparing the first PCoA axis with the ratio of Prevotella to Bacteroides (Figure S1).

If we repeat our PCoA analysis, but at the OTU level, rather than using the RDP genus assignments, we observe a very different pattern with no separation of the biome types (Figure 5 C&D). The OTU analysis shows the gradient of *Bacteroides* and Clostridiales along the first axis, with most of the Clostridiales falling within the area of overlap. Using the V3–V5 OTUs the Prevotellaceae samples fell completely within the area of overlap between *Bacteroides* and Clostridiales with no segregation at all. Using the V1–V3 data, the Prevotellaceae fall exactly along the edge of the *Bacteroides* and Clostridiales, but with no clear separation between the two types. In the V3–V5 data there are 70 distinct *Bacteroides* OTUs and 437 distinct Clostridiales OTUs. By counting these as separate phylotypes, it may be that the community distances within these biome types are too great and the community distances between the biome types too small, combined with the existence of a rich diversity of other OTUs and taxa present in both types precluding adequate PCoA clustering at the OTU level. The OTU data, therefore, appears to report a level of individual discrimination between subjects that confounds stool biome types that are more apparent at a less discriminating level of taxonomic resolution. This observation is consistent with a recent suggestion that the appropriate level of taxonomic resolution be explicitly considered in the analysis of metagenomic data [12] and suggests that there is considerable individual variation between stool samples.

Vaginal samples by contrast appeared to have at least three potential V3–V5 biome types, and at least 4 V1–V3 biome types that do not overlap (Figure 5 D&E). The three vaginal sampling locations (mid-vagina, vaginal introitus, and posterior fornix) yielded almost exactly the same results. The rRNA hypervariable regions, V3–V5 and V1–V3 provided a somewhat different perspective. With the V3–V5 tag data, greater than 90% of the samples were dominated by a single *Lactobacillus* OTU (93% in mid-vagina and 91% in both the vaginal introitus and the posterior fornix). A single Bifidobacteriaceae OTU was the most abundant OTU in about 5% of samples and the remaining samples had several other OTUs representing different taxa (including *Atopobium, Prevotella, Propionobacterium* and Clostridiales) as the most abundant. The dominant V3–V5 *Lactobacillus* OTU includes sequences with perfect BLAST matches to several different species including *L. crispatus, L. iners, L. gasseri,* as well as *L. acidophilus, L. amylovorus, L. kalixensis, L.gallinarum, L. johnsonii,* among others.

Sequencing with V1–V3 separates the vaginal *Lactobacillus* into three separate OTUs likely representing *L. crispatus* (OTU #3), *L. iners* (OTU #6), and *L. gasseri* (OTU #9) (based on perfect match BLAST results of the most abundant sequence to the NCBI nt database). These OTUs correspond to the dominant *Lactobacillus* species and biome types identified by Zhou et al [13] and Ravel et al [14] as Groups I (OTU #3), II (OTU #9), and III (OTU #6) and one dominated by non-Lactobacillus OTUs, including *Prevotella* and *Atopobium*, Group IV. Interestingly, the V1–V3 tags while better at differentiating amongst the *Lactobacillus* species, were not effective in detecting the Bifidobacteriaceae seen in the V3–V5 tags. About 60% of the subjects were dominated by the *Lactobacillus* OTU #3 (60% in the mid-vagina, 64% at the posterior fornix and 61% at the vaginal introitus), 20% were dominated by *Lactobacillus* OTU #6 (25%, 19%, and 21%) and 13% (13%, 12%, 13%) by *Lactobacillus* OTU #9, with the remaining ∼5% dominated by other OTUs and taxa.

### Estimated Total Richness

**Figure 6:**
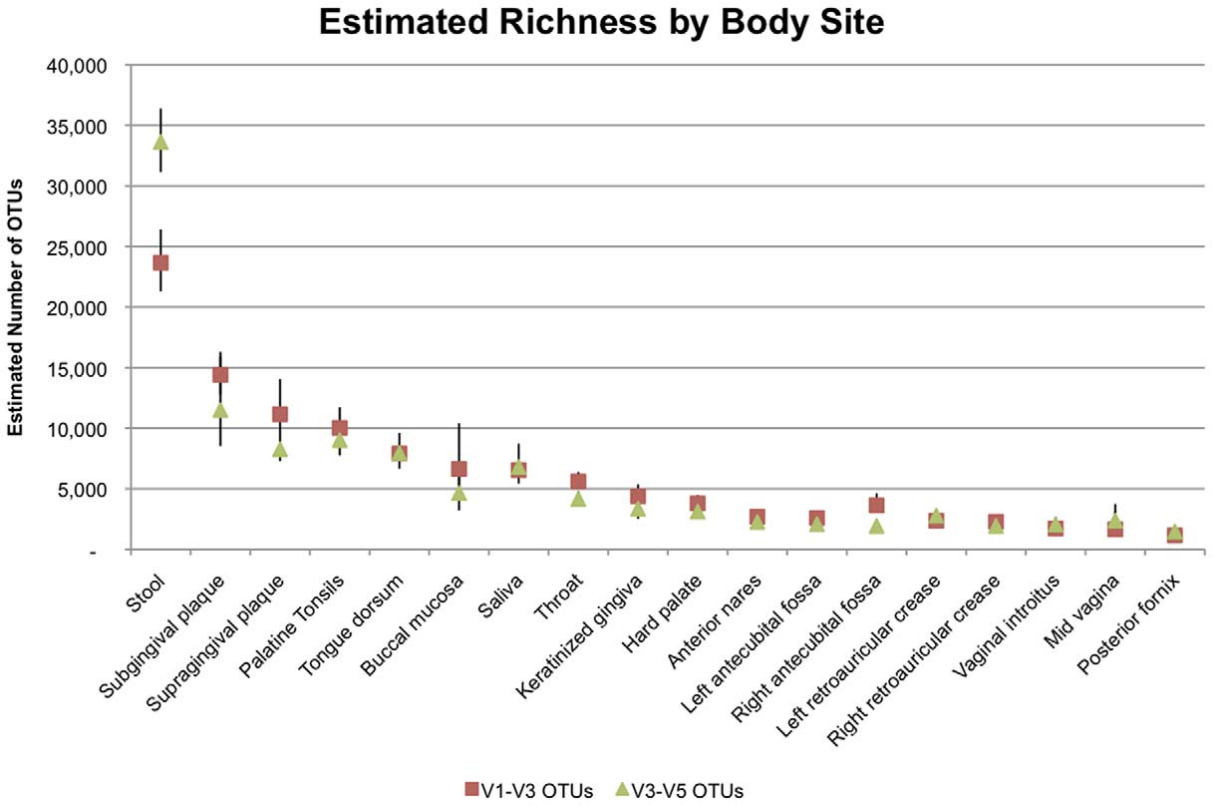
OTU richness estimates for each body site. Estimated richness calculated using CatchAll [16] with both the V1–V3 and the V3–V5 tag data for each body site. Bars represent the upper and lower confidence bounds provided by Catchall. Both sets of rRNA tags provided similar estimates. The stool samples showed the most richness with the oral samples having the next greatest richness, followed by the skin and vaginal samples.

The estimated total richness of the microbial communities, as defined by total number of OTUs expected with complete sampling of the subject population, varies markedly between body sites (See Table 2 and Figure 6). The stool has the highest estimated richness at 33,627 expected V3–V5 OTUs (23,665 V1–V3 OTUs), followed by the oral sites with estimates of richness from 3,125–11,501 V3–V5 OTUs (3,793–14,410 V1–V3 OTUs), and then the anterior nares, skin and vagina sites have richness estimates between 1400 and 2800 V3–V5 OTUs (1100–3700 V1–V3 OTUs). While the richness estimates calculated from the V1–V3 data are on average moderately higher, the body sites maintain a similar ordering of relative richness. The different skin sites have similar richness estimates as do the different vaginal sites. The oral sites, however, have a broader range of estimated richness. By combining samples across subjects and body sites, we can estimate the number of different OTUs that may be expected for the human population sampled. Combining data across all body sites and subjects, we estimate the V3–V5 richness for the female microbiome to be 51,373 (V1–V3∶42,391) and the male V3–V5 richness to be 48,388 (V1–V3∶39,782). These estimates are about 40% of the sum of the individual richness estimates by body site, which implies that more than half of the subgenus OTUs are present in more than one location on the body, although much of this may be due to OTUs appearing in multiple oral sites.

**Table 2:** Richness estimates for each body site.

	V1–V3	V3–V5
**Saliva**	6,546 (5789–7453.1)	6,801 (5421–8718.9)
**Supragingival plaque**	11,154 (9028–14059.4)	8,254 (7280–9420.8)
**Subgingival plaque**	14,410 (12800–16301.3)	11,501 (8533–15930.1)
**Keratinized gingiva**	4,387 (3639–5375.7)	3,352 (2509–4674.4)
**Tongue dorsum**	7,910 (7080–8895.2)	7,947 (6663–9613.9)
**Hard palate**	3,793 (3262–4473.6)	3,125 (2642–3766.9)
**Buccal mucosa**	6,635 (4470–10402.8)	4,650 (3212–7075.7)
**Palatine Tonsils**	10,023 (8647–11724.5)	9,020 (7751–10590.8)
**Throat**	5,601 (4948–6396.8)	4,154 (3633–4806.4)
**Anterior nares**	2,693 (2412–3059.9)	2,264 (1997–2611.8)
**Stool**	23,665 (21299–26411.4)	33,627 (31147–36391.1)
**Left Antecubital fossa**	2,589 (2266–3047.7)	2,091 (1906–2331)
**Right Antecubital fossa**	3,632 (2998–4612.2)	1,933 (1778–2135.6)
**Left Retroauricular crease**	2,363 (2040–2816.9)	1,933 (1761–2150.7)
**Right Retroauricular crease**	2,278 (2000–2656.3)	2,785 (2520–3109.5)
**Mid-vagina**	1,653 (1029–3150.1)	2,379 (1622–3748.7)
**Posterior fornix**	1,151 (872–1594.4)	1,466 (1228–1786.6)
**Vaginal introitus**	1,722 (1293–2446.3)	2,062 (1646–2687.3)

Estimated number of species for each body site using both the V1–V3 and the V3–V5 tags computed with CatchAll. Numbers in parentheses are upper and lower confidence limits. The stool samples have the highest estimate of total richness, followed by the oral sites, particularly the plaque and tonsils. The skin and the vaginal sites have the lowest estimated richness.

### Patterns in the Healthy Human Microbiome Within Genera

Clustering 16S tags into OTUs at the 3% level often differentiates groups of organisms more specifically than the genus level taxonomy. Current bacterial taxonomy is limited such that even with full length rRNA sequences, most organisms cannot be identified to species, and often not even as far as the genus level. The most commonly used tool for assigning taxonomy, the RDP Classifier [4], does not assign taxonomic names below the genus level. OTUs based on 16S tag sequencing, however, can often distinguish between organisms within a single genus that may represent species or strain level taxonomy or simply subgroups of organisms within a given genus. These sub-genus OTUs can reveal patterns not seen at the genus or higher taxonomic levels. While subgenus diversity within rare OTUs may be important, especially when their functions are combined across OTUs [8], the sampling depth of this data is insufficient to support the exploration of rare OTUs, and we only used OTUs containing a minimum of 100 sequences. Although our analyses here focus on the V3–V5 sequencing because of the greater amount of data available, in many cases the V1–V3 OTUs have better discerning power at the sub-genus level, as demonstrated by the ability of the V1–V3 tags to highlight three different *Lactobacillus* OTUs in the vaginal samples (see *Presence of Biome Types*, above).

**Figure 7:**
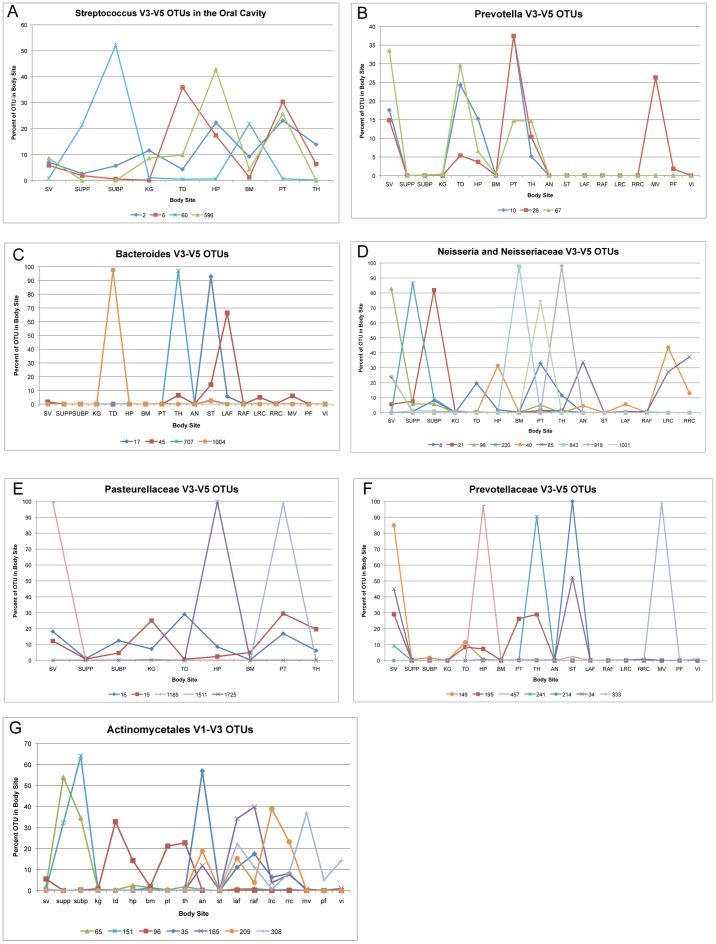
Body site preference of distinct OTUs from individual taxa. The use of genus or family names alone can imply a broad colonization of one organism across body sites. Using 3% OTUs, we can discern a high degree of site specialization of distinct organisms within these taxonomic groups, especially within the oral cavity. Not all OTUs assigned to these taxa were graphed, and only OTUs with greater than 100 tags were included. The area under each curve sums to 100% of the occurrence of that OTU across the body sites. Body site labels are: saliva (sv), supragingival plaque (supp), subgingival plaque (subp), keratinized gingiva (kg), tongue dorsum (td), hard palate (hp), buccal mucosa (bm), palatine tonsils (pt), throat (th), anterior nares (an), stool (st), left and right antecubital fossae (laf, raf), left and right retroauricular creases (lrc, rrc), mid-vagina (mv), posterior fornix (pf), and vaginal introitus (vi).

Of the top most abundant genera in the V3–V5 tag data: *Streptococcus*, *Propionibacterium*, *Lactobacillus*, *Prevotella*, *Bacteroides*, *Corynebacterium*, *Fusobacterium*, *Pasteurella*, *Veillonella* and *Neisseria*, all but *Propionibacterium* showed distinct differences in body site preference at the OTU level. This pattern is repeated in many other taxa. As illustrated in Figure 7, different OTUs within a single genus can have markedly different relative abundances in different body sites. For instance, *Streptococcus*, the most abundant genus in the dataset appears to have at least four different V3–V5 OTUs appearing in the oral sites (Figure 7A), with OTU #2 and #596 most abundant in the hard palate and palatine tonsils. Both OTU #2 and OTU #596 represent sequences matching many different members of the Streptococcus Mitis group (See Table S4 for OTU species assignments). OTU #60 (*S. mutans*) was more abundant in the supra- and sub-gingival plaque and in the buccal mucosa, and OTU #6 (*Streptococcus sp.*) was most abundant on the tongue but also on the hard palate, tonsils. Within the *Prevotella* genus 79 V3–V5 OTUs were discovered. It may be that many of these are a finer differentiation of organisms that have a natural 16S variation >3% or that there are multiple copies of the 16S gene within the genome. The three most abundant of these OTUs: #10 (*P. melaninogenica*), #26 (*P. pallens*), #67 (*P. nanceinensis*) all appear preferentially in the oral cavity but with slightly different abundance patterns between the sites. Only OTU #26 appeared in high numbers in the mid-vagina (Figure 7B). All three of them appear more commonly in saliva, tongue dorsum, hard palate palatine tonsils and the throat, but are distinctly rare in the subgingival and supragingival plaque, keratinized gingiva and the buccal mucosa, as well as the nares, stool and skin. Many of the other *Prevotella* OTUs followed the same patterns as these three.

Four of the twenty *Bacteroides* V3–V5 OTUs demonstrated highly specific body site preferences (Figure 7C). Despite both having taxonomic best matches to *B. vulgatus* and *B. dorei*, OTU #17 appeared almost exclusively in the stool and OTU #707 almost exclusively in the throat. OTU #1004 (*Bacteroides sp.*) appeared almost exclusively on the tongue, but OTU #45 (*B. stercoris*) was found in the throat, stool, left antecubital fossa and mid-vagina (Figure 7B). The seven subjects with the highest abundance of OTU #45 in the left antecubital fossa, accounting for most of the normalized antecubital fossa abundance did not have samples from the right antecubital fossa with adequate tags to be included in the study (less than 1000 reads), therefore no conclusions should be drawn about the left vs. right antecubital fossa and the *Bacteroides* OTU #45.

The genus *Corynebacterium* had at least 8 V3–V5 OTUs with five different profiles: OTU #15 (*C. matruchotii*) was present almost exclusively in the supragingival plaque, OTU #12 (*Clostridium sp.)* was predominantly present in the anterior nares, OTU #188 (*C. argentoratense*) mostly in saliva and to a lesser extent the hard palate, OTU #101 (*Clostridium sp.*) primarily in the skin and the mid-vagina, and OTU #418 (*C. glucuronolyticum*) in the mid-vagina and posterior fornix. Three of the most abundant *Fusobacterium* OTUs OTU #523 (*Fusibacterium sp., Filifactor alocis*), OTU #738 (*Fusibacterium sp., Filifactor alocis*), and OTU #9 (*F. periodonticum)* were present in moderate to high abundance in the tonsils. We also found OTU #523 in the plaque, OTU #738 on the tongue, but OTU #9 was more cosmopolitan appearing in the plaque, on the tongue, in the throat and to a small extent in the mid-vagina.

OTUs can also be used to differentiate sequences whose taxonomy cannot be ascertained even to the genus level, either because the tags themselves cannot be assigned a genus-level taxonomy, or because the tags within an OTU are assigned to different taxa confounding the taxonomic assignment of the OTU. In addition to OTUs belonging to the genus *Neisseria* which included at least four OTUs with distinct locational patterns, peaking in saliva (OTU #98, *Neisseria sp.*), supragingival plaque (OTU #220, *N. bacilliformis*), subgingival plaque (OTU #21, *Neisseria sp,* and *Morococcus cerebrosus*), and the tongue, tonsils and throat (OTU # 8, *Neisseria sp.*), OTUs classifying only to the family level as Neisseriaceae (which could not be further classified with BLAST) peaked in the buccal mucosa (OTU #843), the tonsils (OTU #1001), and the throat (OTU #918) and two more were present in the retroauricular crease, one of which was also on the hard palate (OTUs #40) and the other in the anterior nares (OTU #85) (Figure 7D). While the genera within the Pasteurellaceae family did not further separate into subgenus V3–V5 OTUs, the OTUs classified only to the family level did (Figure 7E), with three OTUs exclusive to a single body site: saliva (OTU #1511), hard palate (OTU #1725), and palatine tonsils (OTU #1185). Two additional OTUs #16 (*Haemophilus parainfluenzae*) and #19 (*Haemophilus haemolyticus*) were each present across most of the oral sites. None of these OTUs were found above trace levels in the nares, stool, skin, or vagina. V3–V5 OTUs assigned to the Prevotellaceae family also showed distinct body site preferences. Five of the most common Prevotellaceae OTUs appeared almost exclusively in a single body site: OTU #214 in the stool, OTU #241 in the throat, OTU #149 in the saliva, OTU #333 (*P. melaninogenica*) in the mid-vagina, and OTU #457 on the hard palate (Figure 7F). OTU #34 was split between the saliva and stool, and a seventh Prevotellaceae OTU, #195, showed more generalization, appearing in the saliva, tonsils and throat, and in lower abundances on the tongue and hard palate. Interestingly, only OTU #333 had best BLAST hits with species level taxonomy assignments. At the order level, V1–V3 OTUs identified only as Actinomycetales included seven OTUs with distinct patterns, with OTUs #65 (*C. durum*) and 151 (*Actinomyces sp.*) preferentially colonizing the subgingival and supragingival plaque, OTU #96 (*Actinomyces gravenitzii*) in several places in the oral cavity, especially the tongue, hard palate, tonsils and throat, OTUs #35 (Actinomycetales), #209 (*Corynebacterium kroppenstedtii*), and #165 (Actinomycetales) in the anterior nares and skin sites, and OTU #308 (*Mycobacterium sp.*) on the skin and in the vagina (Figure 7G).

## Discussion

This overview study is part of a more extensive project providing the largest survey to date of the microbiota that live on and in the healthy human body. We found very few OTUs of the 16S gene (V1–V3 and V3–V5 regions) present in all subjects sampled. Many of the sample sequence sets were relatively small and could not fully reflect the richness of organisms present in the given sample. We therefore removed all samples with less than 1,000 reads and defined the core microbiome as those OTUs that were present in 95% of the samples. By this definition, the oral sites have the highest number of core OTUs, ranging from 3 to 16 followed by the stool and nares, then by the skin and vaginal sites (Table 1). The OTU identifiers and representative sequences for all OTUs considered part of the 95% core microbiome are included in the Supporting Information. The estimated richness of OTUs varies markedly across body sites with the stool being the richest followed by the sites of the oral cavity and then the skin and vaginal samples having the lowest estimated richness.

Although many OTUs were prevalent in specific body locations across subjects the relative abundance of these OTUs varied greatly between subjects and samples. For all OTUs occurring in 10 or more samples from a particular body site and having a relative abundance of 30% or more in at least one sample, the variation in relative abundance varied across samples for that body site by at least two orders of magnitude and frequently 3 or 4 orders of magnitude. The range in abundance of OTUs means that for full comparisons across samples we need an adequate depth of sampling that can detect OTUs at a relative abundance of 1 in 10,000 or less.

We examined the stool and vaginal body sites to look for biome types – recurring and distinct assemblages of microbial communities. Arumugam *et al*. [8] reported specific enterotypes: distinct gut microbial communities, (also referred to as biome types), in different groups of individuals. They found three stool biome types: one dominated by *Bacteroides*, the second by *Prevotella*, and the third by Closteridiales (16S and Illumina metagenomic tags) or *Ruminococcus* (Sanger metagenomic sequences). The results of this study were partially supported by Wu et al [11] who analyzed 98 16S tags and argued that the continuum of samples they observed was best described by 2 biome types, with the *Ruminococcus* combined with the *Bacteroides* group but along a continuum between these two types rather than as distinct, and *Prevotella* as the second. At the genus (order) level, the HMP cohort is consistent with Wu *et al*. [11], both in the assessment of two rather than three biome types (*Bacteroides*/Clostridiales and *Prevotella*) although the data are not well separated and represent a gradient of the microbial communities including and between these biome types rather than distinct biome types (see Figure 1 and Wu *et al.* [11] Figure S5). Repeating the PCoA analysis using OTUs, still did not demonstrate distinct clusterings (Figure 5). These stool biome types do not represent distinct or exclusive groups, but rather they express the combined gradients of the three most abundant taxa with substantial overlap between the Bacteroides and Clostridiales stool communities and less overlap between these two and the Prevotella communities.

The vaginal samples did separate into biome types. The choice of rRNA hypervariable region, however, affected the ability to differentiate the organisms involved, but all three vaginal sites (vaginal introitus, mid-vagina, and posterior fornix) displayed the same patterns. We found upwards of 90% of our subjects were dominated by *Lactobacillus*. While the V3–V5 tags could not differentiate the *Lactobacillus*, the V1–V3 tags separated the *Lactobacilli* into three distinct OTUs, with around 60% of subjects dominated by OTU #3, 21% by OTU #6 and 12% by OTU #9 in each vaginal site. The V3–V5 tags, on the other hand, pointed at a Bifidobacteriaceae OTU as dominating in about 5% of case, although this taxon was not well discovered by the V1–V3 tags, either because of a mismatch of the primers for amplification or because of an inability to differentiate the taxon in that region of the rRNA gene.

Multiple bacteria within the same genus show a high degree of niche specificity between body sites, especially between sites of the oral cavity (Figure 7A–G). In the absence of consistent species-level taxonomy, the OTUs provide critical differentiation between organisms within the same genera or higher classification. In particular, we examined the 10 most abundant genera in our subjects and found that all but one had multiple OTUs with distinct preferences across the body sites. Most of these were present within the oral cavity where different OTUs from the same genus show clear preferences for often only one or two of the nine oral sites. This was true for *Bacteroides*, *Prevotella*, *Corynebacterium*, *Fusobacterium*, *Pasteurella*, *Veillonella* and *Neisseria*. These patterns of clear niche differentiation were repeated in several cases where the taxonomic name of the OTU could only be resolved to the family or order level, including Neisseriaceae, Pasteurellaceae, Prevotellaceae, and Actinomycetales. Our results highlight the importance of using taxonomic-independent, sequenced-based methods such as 16S OTUs as well as taxonomy when assessing the diversity and niche selection of human microbial communities. Our results correlated well between the V1–V3 and the V3–V5 regions of the 16S gene, with some taxa resolving better with V1–V3 and others with the V3–V5. As with all 16S tag sequencing projects, the specific richness and diversity results should be compared with other results using the same 16S region, and the presence of primer bias should not be discounted.

## Methods

The human data used in this manuscript was provided by the NIH Human Microbiome Project. All subjects signed a written consent form. The sampling of human tissue was performed at several study sites. The Institutional Review Boards at each study site reviewed and approved the protocol, informed consent and other study documents. They obtained Certificates of Confidentiality intended to protect against the compelled disclosure in legal proceedings of the participants’ identities. The study sites are: Baylor College of Medicine, Washington University in St. Louis, St. Louis University, and the University of Texas Health Science Center at Houston. All subjects were screened for general health before inclusion, and signed a written consent form. Full screening methods and consent information are reported in Aagard *et al*. [2].

The quality filtering and trimming, chimera removal, taxonomic assignments and OTU clustering of the high quality V1–V3 and V3–V5 pyrosequencing tags provided by the Human Microbiome Project were performed by Pat Schloss using mothur [15]. In the few cases where there were multiple visits or repeated sequencing, we included only the first visit or replicate. To minimize the effect of undersampling while maintaining as broad a dataset as possible, we only included samples containing 1,000 or more tags. Sequence data for the HMP have been made public through the Data Analysis and Coordination Center, http://www.hmpdacc.org.

Prevalence was calculated as the percent of samples containing a given OTU and abundance as the count of reads (or percent of sample) belonging to a particular OTU. We calculated estimated richness using CatchAll [16]. We calculated the community distances and performed the principle coordinates analyses (PCoA) using mothur. All samples were normalized to relative abundance by dividing the read counts by the sample size before comparing across samples and community distances were calculated using Morisita-Horn.

To determine the taxa for the stool samples at the OTU level, we calculated the normalized relative abundance for each OTU and each subject. We then sorted the OTUs in rank order of summed relative abundance across all subjects. We summed the relative abundances of all OTUs in a sample belonging to either the genus *Bacteroides*, the order Clostridiales as per Arumugan et al [8]. The most prevalent OTU containing *Prevotella* sequences was assigned the consensus taxonomy of Prevotellaceae, so we summed Prevotellaceae OTUs rather than just *Prevotella* OTUs. Samples were assigned to one of these three groups depending on which group was most abundant. If any other OTU outside of these three groups was more abundant, the sample was reassigned to the “Other” group. We used Morisita-Horn distance metric and mothur for Figures 5C and 5D. We repeated the stool OTU–level analysis with UniFrac with very similar results (not shown). A similar procedure was used for the vaginal biome types. The OTU having the highest relative abundance determined the biome type, either OTU #3 (*Lactobacillus*) or OTU #47 (Bifidobacteriaceae) or other for the V3–V5, and OTU #3, 6, 9 or other for the V1–V3 data.

We assessed the relative abundance of sub-genus OTUs across body sites by normalizing each sample first, summing the relative abundances of each OTU across the body sites, and dividing the total relative abundance of each OTU in each body site, by the total relative abundance of each OTU across body sites. In Figure 7, each OTU will sum to 100% across all body sites. We annotated OTUs in *Patterns in the healthy human microbiome within genera*, by BLASTing the 3 most common sequences in each OTU against the NCBI nt database, excluding uncultured/environmental sequence samples, and compiling all species-level hits matching the best query coverage and percent identity found. These assignments are for the most abundant tags only and do not represent a consensus of all sequences in the OTUs which often include multiple genera, species, or strains.

## Supporting Information

Figure S1 **Bacteroides – Prevotella Gradient.** The ratio of Prevotella to Bacteroides for the V3–V5 data using taxonomy assigned directly to 16S tags, not OTU clustering. The principal coordinate axis 2 (see Figure 5A) provides the most differentiation between these two biome types. The samples display a full spectrum of ratios of Prevotella to Bacteroides from zero (Prevotella not found) to 4.5. Since the biome type is defined by the most abundant of the two taxa in any sample, the break point between the two is when the ratio of taxa equals one and they are equally abundant. The segregation between the two biome types is an artifact of the definition.
(TIF)

Table S1 **Counts of patients included, total number of 16S tags (sequence reads) and OTUs found for both the V1–V3 and the V3–V5 regions, for data passing the read quality and sample size requirements (see Methods).**
(DOCX)

Table S2 **Number of Core OTUs present at different prevalence thresholds.** Data represent the number of core OTUs found using either the V1–V3 or the V3–V5 regions of the 16S rRNA gene. Values are reported for OTUs present in 100%, 95%, 90%, 75%, and 50% of samples. The first number in each cell is the number of core OTUs for that body site, 16S region and prevalence. The second number is the percent of all sample tags for that body site and 16S region represented by the core OTUs.
(DOC)

Table S3 **The OTU-level consensus taxonomy for each of the OTUs identified as core in a body site at the 95% level.**
(DOC)

Table S4 **Example species-level taxonomy for selected OTUs reported in the section** ***Patterns in the healthy human microbiome within genera*** **.** Sequence tags were initially identified with RDP only to genus or higher, and then each OTU was assigned a consensus of all taxa present. These example species-level taxa assignments are based on BLAST to nt, excluding uncultured and environmental sample sequences for the three most abundant sequences in each OTU. All taxa from sequences matching the best query coverage and percent identity were considered most likely species assignments.
(DOCX)
