# Supplementary figures and images for: A Core Human Microbiome as Viewed through 16S rRNA Sequence Clusters
Source: PLoS One. 2012 Jun 13;7(6):e34242. doi: 10.1371/journal.pone.0034242 (PMC3374614; doi:10.1371/journal.pone.0034242)

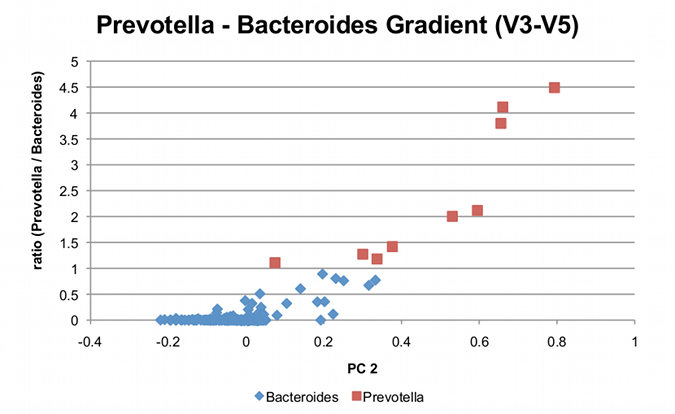

Supplement: Figure S1 — Bacteroides – Prevotella Gradient. The ratio of Prevotella to Bacteroides for the V3–V5 data using taxonomy assigned directly to 16S tags, not OTU clustering. The principal coordinate axis 2 (see Figure 5A) provides the most differentiation between these two biome types. The samples display a full spectrum of ratios of Prevotella to Bacteroides from zero (Prevotella not found) to 4.5. Since the biome type is defined by the most abundant of the two taxa in any sample, the break point between the two is when the ratio of taxa equals one and they are equally abundant. The segregation between the two biome types is an artifact of the definition. (TIF) [file pone.0034242.s001.tif]
