# Supplementary material for: A Core Human Microbiome as Viewed through 16S rRNA Sequence Clusters
Source: PLoS One. 2012 Jun 13;7(6):e34242. doi: 10.1371/journal.pone.0034242 (PMC3374614; doi:10.1371/journal.pone.0034242)
Supplement: Table S1 — Counts of patients included, total number of 16S tags (sequence reads) and OTUs found for both the V1–V3 and the V3–V5 regions, for data passing the read quality and sample size requirements (see Methods). (DOCX) [file pone.0034242.s002.docx]

Table S1

|  | **Counts for V1-V3 region** | | | **Counts for V3-V5 region** | | |
| --- | --- | --- | --- | --- | --- | --- |
| **Body Site** | **Patients** | **Tags** | **OTUs** | **Patients** | **Tags** | **OTUs** |
| Anterior nares | 107 | 584857 | 1323 | 156 | 633582 | 915 |
| Buccal mucosa | 114 | 703212 | 2025 | 198 | 917506 | 898 |
| Hard palate | 112 | 623725 | 1741 | 190 | 929286 | 912 |
| Keratinized gingiva | 117 | 745588 | 1545 | 206 | 951475 | 857 |
| L_Antecubital fossa | 83 | 401925 | 1323 | 67 | 212689 | 1039 |
| L_Retroauricular crease | 126 | 1113183 | 1077 | 173 | 904213 | 872 |
| Mid vagina | 60 | 377823 | 606 | 95 | 586365 | 536 |
| Palatine Tonsils | 119 | 638096 | 3683 | 204 | 1006194 | 1633 |
| Posterior fornix | 59 | 359615 | 428 | 95 | 635668 | 400 |
| R_Antecubital fossa | 87 | 423184 | 1591 | 70 | 219988 | 1044 |
| R_Retroauricular crease | 127 | 960650 | 1091 | 183 | 941661 | 1033 |
| Saliva | 99 | 442633 | 2341 | 181 | 753798 | 1399 |
| Stool | 118 | 652467 | 6050 | 209 | 1122324 | 5391 |
| Subgingival plaque | 119 | 645234 | 4216 | 204 | 951603 | 1672 |
| Supragingival plaque | 121 | 671703 | 3851 | 205 | 952579 | 1587 |
| Throat | 110 | 538845 | 2343 | 192 | 853629 | 1136 |
| Tongue dorsum | 119 | 648334 | 3651 | 205 | 1137210 | 1503 |
| Vaginal introitus | 56 | 366935 | 741 | 88 | 504874 | 603 |
