# Supplementary material for: A Core Human Microbiome as Viewed through 16S rRNA Sequence Clusters
Source: PLoS One. 2012 Jun 13;7(6):e34242. doi: 10.1371/journal.pone.0034242 (PMC3374614; doi:10.1371/journal.pone.0034242)
Supplement: Table S2 — Number of Core OTUs present at different prevalence thresholds. Data represent the number of core OTUs found using either the V1–V3 or the V3–V5 regions of the 16S rRNA gene. Values are reported for OTUs present in 100%, 95%, 90%, 75%, and 50% of samples. The first number in each cell is the number of core OTUs for that body site, 16S region and prevalence. The second number is the percent of all sample tags for that body site and 16S region represented by the core OTUs. (DOC) [file pone.0034242.s003.doc]

Table S2

|  | **100%** | | **95%** | | **90%** | | **75%** | | **50%** | |
| --- | --- | --- | --- | --- | --- | --- | --- | --- | --- | --- |
|  | **V1-V3** | **V3-V5** | **V1-V3** | **V3-V5** | **V1-V3** | **V3-V5** | **V1-V3** | **V3-V5** | **V1-V3** | **V3-V5** |
| **Saliva** | 0 / 0% | 7 / 26% | 12 / 28% | 22 / 41% | 23 / 39% | 29 / 44% | 44 / 47% | 44 / 48% | 79 / 52% | 75 / 52% |
| **Supragingival plaque** | 5 / 12% | 4 / 19% | 13 / 30% | 15 / 38% | 20 / 40% | 22 / 45% | 32 / 45% | 32 / 50% | 56 / 49% | 57 / 57% |
| **Hard palate** | 6 / 44% | 8 / 47% | 14 / 54% | 16 / 58% | 20 / 59% | 21 / 61% | 36 / 65% | 31 / 63% | 60 / 68% | 51 / 68% |
| **Palatine Tonsils** | 8 / 19% | 3 / 16% | 14 / 27% | 16 / 42% | 23 / 35% | 21 / 46% | 38 / 39% | 32 / 49% | 68 / 44% | 61 / 57% |
| **Tongue dorsum** | 6 / 26% | 5 / 31% | 15 / 44% | 13 / 51% | 21 / 49% | 22 / 59% | 33 / 57% | 30 / 62% | 49 / 60% | 43 / 65% |
| **Throat** | 4 / 16% | 3 / 15% | 15 / 32% | 13 / 36% | 22 / 37% | 21 / 42% | 34 / 41% | 33 / 45% | 57 / 45% | 54 / 51% |
| **Buccal mucosa** | 3 / 40% | 6 / 50% | 11 / 50% | 11 / 59% | 15 / 52% | 17 / 63% | 25 / 57% | 24 / 64% | 54 / 61% | 47 / 68% |
| **Subgingival plaque** | 1 / 4% | 0 / 0% | 10 / 20% | 7 / 18% | 16 / 23% | 19 / 29% | 36 / 35% | 37 / 36% | 67 / 40% | 64 / 44% |
| **Keratinized gingiva** | 2 / 36% | 1 / 32% | 3 / 39% | 7 / 56% | 6 / 43% | 8 / 56% | 14 / 53% | 12 / 69% | 33 / 64% | 19 / 72% |
| **Anterior nares** | 0 / 0% | 2 / 17% | 3 / 21% | 4 / 32% | 4 / 22% | 4 / 32% | 8 / 25% | 6 / 34% | 18 / 26% | 18 / 37% |
| **Stool** | 2 / 1% | 1 / 3% | 7 / 6% | 5 / 8% | 11 / 8% | 7 / 9% | 22 / 11% | 20 / 11% | 47 / 13% | 50 / 15% |
| **Right Antecubital fossa** | 1 / 9% | 0 / 0% | 1 / 9% | 3 / 13% | 2 / 11% | 3 / 13% | 7 / 15% | 8 / 15% | 16 / 17% | 29 / 23% |
| **Left Antecubital fossa** | 1 / 10% | 0 / 0% | 1 / 10% | 2 / 8% | 2 / 13% | 3 / 10% | 5 / 15% | 10 / 12% | 15 / 19% | 34 / 21% |
| **Left Retroauricular crease** | 1 / 24% | 1 / 27% | 2 / 35% | 2 / 34% | 3 / 37% | 2 / 34% | 5 / 40% | 4 / 36% | 8 / 41% | 11 / 39% |
| **Right Retroauricular crease** | 1 / 21% | 1 / 23% | 2 / 30% | 2 / 29% | 2 / 30% | 3 / 31% | 6 / 35% | 4 / 31% | 8 / 36% | 8 / 33% |
| **Posterior fornix** | 0 / 0% | 0 / 0% | 0 / 0% | 1 / 38% | 0 / 0% | 1 / 38% | 1 / 29% | 1 / 38% | 4 / 43% | 2 / 39% |
| **Mid vagina** | 0 / 0% | 0 / 0% | 0 / 0% | 1 / 41% | 0 / 0% | 1 / 41% | 1 / 26% | 1 / 41% | 10 / 42% | 4 / 42% |
| **Vaginal introitus** | 0 / 0% | 0 / 0% | 0 / 0% | 1 / 37% | 0 / 0% | 1 / 37% | 2 / 28% | 2 / 37% | 12 / 43% | 11 / 44% |
