# Supplementary material for: A Core Human Microbiome as Viewed through 16S rRNA Sequence Clusters
Source: PLoS One. 2012 Jun 13;7(6):e34242. doi: 10.1371/journal.pone.0034242 (PMC3374614; doi:10.1371/journal.pone.0034242)
Supplement: Table S3 — The OTU-level consensus taxonomy for each of the OTUs identified as core in a body site at the 95% level. (DOC) [file pone.0034242.s004.doc]

Table S3

| **Body Site** | **16S Region** | **OTU #** | **Taxonomy** |
| --- | --- | --- | --- |
| Anterior Nares | V1-V3 | 4 | Firmicutes;Bacilli;Bacillales;Staphylococcaceae;Staphylococcus |
|  |  | 1 | Actinobacteria;Actinobacteria;Actinomycetales;Propionibacteriaceae;Propionibacterium |
|  |  | 11 | Actinobacteria;Actinobacteria;Actinomycetales;Corynebacteriaceae;Corynebacterium |
|  | V3-V5 | 1 | Actinobacteria;Actinobacteria;Actinomycetales;Propionibacteriaceae;Propionibacterium |
|  |  | 5 | Firmicutes;Bacilli;Bacillales;Staphylococcaceae;Staphylococcus |
|  |  | 12 | Actinobacteria;Actinobacteria;Actinomycetales;Corynebacteriaceae;Corynebacterium |
|  |  | 2 | Firmicutes;Bacilli;Lactobacillales;Streptococcaceae;Streptococcus |
| Buccal mucosa | V1-V3 | 2 | Firmicutes;Bacilli;Lactobacillales;Streptococcaceae;Streptococcus |
|  |  | 8 | Firmicutes;Bacilli;Bacillales;Staphylococcaceae;Gemella |
|  |  | 7 | Firmicutes;Clostridia;Clostridiales;Veillonellaceae;Veillonella |
|  |  | 5 | Firmicutes;Bacilli;Lactobacillales;Streptococcaceae;Streptococcus |
|  |  | 10 | Actinobacteria;Actinobacteria;Actinomycetales;Micrococcaceae;Rothia |
|  |  | 27 | Fusobacteria;Fusobacteria;Fusobacteriales;Fusobacteriaceae;Fusobacterium |
|  |  | 19 | Firmicutes;Bacilli;Lactobacillales;Carnobacteriaceae;Granulicatella |
|  |  | 22 | Bacteroidetes;Bacteroidia;Bacteroidales;Porphyromonadaceae;Porphyromonas |
|  |  | 24 | Actinobacteria;Actinobacteria;Actinomycetales;Actinomycetaceae;Actinomyces |
|  |  | 20 | Proteobacteria;Gammaproteobacteria;Pasteurellales;Pasteurellaceae;Haemophilus |
|  |  | 38 | Fusobacteria;Fusobacteria;Fusobacteriales;Leptotrichiaceae;Leptotrichia |
|  | V3-V5 | 2 | Firmicutes;Bacilli;Lactobacillales;Streptococcaceae;Streptococcus |
|  |  | 16 | Proteobacteria;Gammaproteobacteria;Pasteurellales;Pasteurellaceae;Pasteurella |
|  |  | 11 | Firmicutes;Bacilli;Bacillales;Staphylococcaceae;Gemella |
|  |  | 6 | Firmicutes;Bacilli;Lactobacillales;Streptococcaceae;Streptococcus |
|  |  | 4 | Firmicutes;Clostridia;Clostridiales;Veillonellaceae;Veillonella |
|  |  | 9 | Fusobacteria;Fusobacteria;Fusobacteriales;Fusobacteriaceae;Fusobacterium |
|  |  | 19 | Proteobacteria;Gammaproteobacteria;Pasteurellales;Pasteurellaceae;Actinobacillus |
|  |  | 7 | Bacteroidetes;Bacteroidia;Bacteroidales;Porphyromonadaceae;Porphyromonas |
|  |  | 13 | Firmicutes;Bacilli;Lactobacillales;Carnobacteriaceae;Granulicatella |
|  |  | 8 | Proteobacteria;Betaproteobacteria;Neisseriales;Neisseriaceae;Kingella |
|  |  | 10 | Bacteroidetes;Bacteroidia;Bacteroidales;Prevotellaceae;Prevotella |
| Hard palate | V1-V3 | 2 | Firmicutes;Bacilli;Lactobacillales;Streptococcaceae;Streptococcus |
|  |  | 5 | Firmicutes;Bacilli;Lactobacillales;Streptococcaceae;Streptococcus |
|  |  | 7 | Firmicutes;Clostridia;Clostridiales;Veillonellaceae;Veillonella |
|  |  | 8 | Firmicutes;Bacilli;Bacillales;Staphylococcaceae;Gemella |
|  |  | 16 | Bacteroidetes;Bacteroidia;Bacteroidales;Prevotellaceae;Prevotella |
|  |  | 29 | Firmicutes;Clostridia;Clostridiales;Lachnospiraceae;Coprococcus |
|  |  | 34 | Actinobacteria;Actinobacteria;Actinomycetales;Actinomycetaceae;Actinomyces |
|  |  | 19 | Firmicutes;Bacilli;Lactobacillales;Carnobacteriaceae;Granulicatella |
|  |  | 13 | Actinobacteria;Actinobacteria;Actinomycetales;Micrococcaceae;Rothia |
|  |  | 20 | Proteobacteria;Gammaproteobacteria;Pasteurellales;Pasteurellaceae;Haemophilus |
|  |  | 12 | Proteobacteria;Betaproteobacteria;Neisseriales;Neisseriaceae;Neisseria |
|  |  | 27 | Fusobacteria;Fusobacteria;Fusobacteriales;Fusobacteriaceae;Fusobacterium |
|  |  | 14 | Proteobacteria;Epsilonproteobacteria;Campylobacterales;Campylobacteraceae;Campylobacter |
|  |  | 38 | Fusobacteria;Fusobacteria;Fusobacteriales;Leptotrichiaceae;Leptotrichia |
|  |  | 2 | Firmicutes;Bacilli;Lactobacillales;Streptococcaceae;Streptococcus |
|  | V3-V5 | 2 | Firmicutes;Bacilli;Lactobacillales;Streptococcaceae;Streptococcus |
|  |  | 6 | Firmicutes;Bacilli;Lactobacillales;Streptococcaceae;Streptococcus |
|  |  | 16 | Proteobacteria;Gammaproteobacteria;Pasteurellales;Pasteurellaceae;Pasteurella |
|  |  | 4 | Firmicutes;Clostridia;Clostridiales;Veillonellaceae;Veillonella |
|  |  | 10 | Bacteroidetes;Bacteroidia;Bacteroidales;Prevotellaceae;Prevotella |
|  |  | 9 | Fusobacteria;Fusobacteria;Fusobacteriales;Fusobacteriaceae;Fusobacterium |
|  |  | 11 | Firmicutes;Bacilli;Bacillales;Staphylococcaceae;Gemella |
|  |  | 14 | Actinobacteria;Actinobacteria;Actinomycetales;Actinomycetaceae;Actinomyces |
|  |  | 13 | Firmicutes;Bacilli;Lactobacillales;Carnobacteriaceae;Granulicatella |
|  |  | 8 | Proteobacteria;Betaproteobacteria;Neisseriales;Neisseriaceae;Neisseria |
|  |  | 19 | Proteobacteria;Gammaproteobacteria;Pasteurellales;Pasteurellaceae;Actinobacillus |
|  |  | 25 | Bacteroidetes;Bacteroidia;Bacteroidales;Bacteroidaceae;Bacteroides |
|  |  | 7 | Bacteroidetes;Bacteroidia;Bacteroidales;Porphyromonadaceae;Porphyromonas |
|  |  | 50 | Firmicutes;Clostridia;Clostridiales;Lachnospiraceae;Johnsonella |
|  |  | 33 | Firmicutes;Clostridia;Clostridiales;Lachnospiraceae;Oribacterium |
|  |  | 18 | Actinobacteria;Actinobacteria;Actinomycetales;Micrococcaceae;Rothia |
| Keratinized gingiva | V1-V3 | 2 | Firmicutes;Bacilli;Lactobacillales;Streptococcaceae;Streptococcus |
|  |  | 8 | Firmicutes;Bacilli;Bacillales;Staphylococcaceae;Gemella |
|  |  | 5 | Firmicutes;Bacilli;Lactobacillales;Streptococcaceae;Streptococcus |
|  | V3-V5 | 2 | Firmicutes;Bacilli;Lactobacillales;Streptococcaceae;Streptococcus |
|  |  | 16 | Proteobacteria;Gammaproteobacteria;Pasteurellales;Pasteurellaceae;Pasteurella |
|  |  | 4 | Firmicutes;Clostridia;Clostridiales;Veillonellaceae;Veillonella |
|  |  | 11 | Firmicutes;Bacilli;Bacillales;Staphylococcaceae;Gemella |
|  |  | 19 | Proteobacteria;Gammaproteobacteria;Pasteurellales;Pasteurellaceae;Actinobacillus |
|  |  | 9 | Fusobacteria;Fusobacteria;Fusobacteriales;Fusobacteriaceae;Fusobacterium |
|  |  | 13 | Firmicutes;Bacilli;Lactobacillales;Carnobacteriaceae;Granulicatella |
| Left antecubital fossa | V1-V3 | 2 | Firmicutes;Bacilli;Lactobacillales;Streptococcaceae;Streptococcus |
|  | V3-V5 | 1 | Actinobacteria;Actinobacteria;Actinomycetales;Propionibacteriaceae;Propionibacterium |
|  |  | 2 | Firmicutes;Bacilli;Lactobacillales;Streptococcaceae;Streptococcus |
| Left auricular crease | V1-V3 | 1 | Actinobacteria;Actinobacteria;Actinomycetales;Propionibacteriaceae;Propionibacterium |
|  |  | 4 | Firmicutes;Bacilli;Bacillales;Staphylococcaceae;Staphylococcus |
|  | V3-V5 | 1 | Actinobacteria;Actinobacteria;Actinomycetales;Propionibacteriaceae;Propionibacterium |
|  |  | 4 | Firmicutes;Bacilli;Bacillales;Staphylococcaceae;Staphylococcus |
| Mid-vagina | V1-V3 | N/A |  |
|  | V3-V5 | 3 | Firmicutes;Bacilli;Lactobacillales;Lactobacillaceae;Lactobacillus |
| Palatine tonsils | V1-V3 | 2 | Firmicutes;Bacilli;Lactobacillales;Streptococcaceae;Streptococcus |
|  |  | 7 | Firmicutes;Clostridia;Clostridiales;Veillonellaceae;Veillonella |
|  |  | 27 | Fusobacteria;Fusobacteria;Fusobacteriales;Fusobacteriaceae;Fusobacterium |
|  |  | 29 | Firmicutes;Clostridia;Clostridiales;Lachnospiraceae;Oribacterium |
|  |  | 38 | Fusobacteria;Fusobacteria;Fusobacteriales;Leptotrichiaceae;Leptotrichia |
|  |  | 14 | Proteobacteria;Epsilonproteobacteria;Campylobacterales;Campylobacteraceae;Campylobacter |
|  |  | 8 | Firmicutes;Bacilli;Bacillales;Staphylococcaceae;Gemella |
|  |  | 19 | Firmicutes;Bacilli;Lactobacillales;Carnobacteriaceae;Granulicatella |
|  |  | 5 | Firmicutes;Bacilli;Lactobacillales;Streptococcaceae;Streptococcus |
|  |  | 16 | Bacteroidetes;Bacteroidia;Bacteroidales;Prevotellaceae;Prevotella |
|  |  | 34 | Actinobacteria;Actinobacteria;Actinomycetales;Actinomycetaceae;Actinomyces |
|  |  | 56 | Firmicutes;Clostridia;Clostridiales;Lachnospiraceae;Catonella |
|  |  | 13 | Actinobacteria;Actinobacteria;Actinomycetales;Micrococcaceae;Rothia |
|  |  | 78 | Actinobacteria;Actinobacteria;Actinomycetales;Actinomycetaceae;Actinomyces |
|  | V3-V5 | 2 | Firmicutes;Bacilli;Lactobacillales;Streptococcaceae;Streptococcus |
|  |  | 4 | Firmicutes;Clostridia;Clostridiales;Veillonellaceae;Veillonella |
|  |  | 16 | Proteobacteria;Gammaproteobacteria;Pasteurellales;Pasteurellaceae;Pasteurella |
|  |  | 9 | Fusobacteria;Fusobacteria;Fusobacteriales;Fusobacteriaceae;Fusobacterium |
|  |  | 6 | Firmicutes;Bacilli;Lactobacillales;Streptococcaceae;Streptococcus |
|  |  | 13 | Firmicutes;Bacilli;Lactobacillales;Carnobacteriaceae;Granulicatella |
|  |  | 10 | Bacteroidetes;Bacteroidia;Bacteroidales;Prevotellaceae;Prevotella |
|  |  | 11 | Firmicutes;Bacilli;Bacillales;Staphylococcaceae;Gemella |
|  |  | 33 | Firmicutes;Clostridia;Clostridiales;Lachnospiraceae;Oribacterium |
|  |  | 14 | Actinobacteria;Actinobacteria;Actinomycetales;Actinomycetaceae;Actinomyces |
|  |  | 50 | Firmicutes;Clostridia;Clostridiales;Lachnospiraceae;Johnsonella |
|  |  | 25 | Bacteroidetes;Bacteroidia;Bacteroidales;Bacteroidaceae;Bacteroides |
|  |  | 8 | Proteobacteria;Betaproteobacteria;Neisseriales;Neisseriaceae;Neisseria |
|  |  | 7 | Bacteroidetes;Bacteroidia;Bacteroidales;Porphyromonadaceae;Porphyromonas |
|  |  | 20 | Fusobacteria;Fusobacteria;Fusobacteriales;Leptotrichiaceae;Leptotrichia |
|  |  | 68 | Firmicutes;Clostridia;Clostridiales;Lachnospiraceae;Catonella |
| Posterior fornix | V1-V3 | N/A |  |
|  | V3-V5 | 3 | Firmicutes;Bacilli;Lactobacillales;Lactobacillaceae;Lactobacillus |
| Right antecubital fossa | V1-V3 | 1 | Actinobacteria;Actinobacteria;Actinomycetales;Propionibacteriaceae;Propionibacterium |
|  | V3-V5 | 1 | Actinobacteria;Actinobacteria;Actinomycetales;Propionibacteriaceae;Propionibacterium |
|  |  | 5 | Firmicutes;Bacilli;Bacillales;Staphylococcaceae;Staphylococcus |
|  |  | 2 | Firmicutes;Bacilli;Lactobacillales;Streptococcaceae;Streptococcus |
| Right retroauricular crease | V1-V3 | 1 | Actinobacteria;Actinobacteria;Actinomycetales;Propionibacteriaceae;Propionibacterium |
|  |  | 4 | Firmicutes;Bacilli;Bacillales;Staphylococcaceae;Staphylococcus |
|  | V3-V5 | 1 | Actinobacteria;Actinobacteria;Actinomycetales;Propionibacteriaceae;Propionibacterium |
|  |  | 5 | Firmicutes;Bacilli;Bacillales;Staphylococcaceae;Staphylococcus |
| Saliva | V1-V3 | 2 | Firmicutes;Bacilli;Lactobacillales;Streptococcaceae;Streptococcus |
|  |  | 7 | Firmicutes;Clostridia;Clostridiales;Veillonellaceae;Veillonella |
|  |  | 27 | Fusobacteria;Fusobacteria;Fusobacteriales;Fusobacteriaceae;Fusobacterium |
|  |  | 14 | Proteobacteria;Epsilonproteobacteria;Campylobacterales;Campylobacteraceae;Campylobacter |
|  |  | 5 | Firmicutes;Bacilli;Lactobacillales;Streptococcaceae;Streptococcus |
|  |  | 16 | Bacteroidetes;Bacteroidia;Bacteroidales;Prevotellaceae;Prevotella |
|  |  | 29 | Firmicutes;Clostridia;Clostridiales;Lachnospiraceae;Oribacterium |
|  |  | 18 | TM7;TM7_genera_incertae_sedis |
|  |  | 51 | TM7;TM7_genera_incertae_sedis |
|  |  | 8 | Firmicutes;Bacilli;Bacillales;Staphylococcaceae;Gemella |
|  |  | 38 | Fusobacteria;Fusobacteria;Fusobacteriales;Leptotrichiaceae;Leptotrichia |
|  |  | 12 | Proteobacteria;Betaproteobacteria;Neisseriales;Neisseriaceae;Neisseria |
|  | V3-V5 | 4 | Firmicutes;Clostridia;Clostridiales;Veillonellaceae;Veillonella |
|  |  | 2 | Firmicutes;Bacilli;Lactobacillales;Streptococcaceae;Streptococcus |
|  |  | 16 | Proteobacteria;Gammaproteobacteria;Pasteurellales;Pasteurellaceae;Pasteurella |
|  |  | 10 | Bacteroidetes;Bacteroidia;Bacteroidales;Prevotellaceae;Prevotella |
|  |  | 9 | Fusobacteria;Fusobacteria;Fusobacteriales;Fusobacteriaceae;Fusobacterium |
|  |  | 6 | Firmicutes;Bacilli;Lactobacillales;Streptococcaceae;Streptococcus |
|  |  | 14 | Actinobacteria;Actinobacteria;Actinomycetales;Actinomycetaceae;Actinomyces |
|  |  | 7 | Bacteroidetes;Bacteroidia;Bacteroidales;Porphyromonadaceae;Porphyromonas |
|  |  | 8 | Proteobacteria;Betaproteobacteria;Neisseriales;Neisseriaceae;Neisseria |
|  |  | 33 | Firmicutes;Clostridia;Clostridiales;Lachnospiraceae;Oribacterium |
|  |  | 25 | Bacteroidetes;Bacteroidia;Bacteroidales;Bacteroidaceae;Bacteroides |
|  |  | 29 | Firmicutes;Clostridia;Clostridiales;Veillonellaceae;Selenomonas |
|  |  | 13 | Firmicutes;Bacilli;Lactobacillales;Carnobacteriaceae;Granulicatella |
|  |  | 19 | Proteobacteria;Gammaproteobacteria;Pasteurellales;Pasteurellaceae;Actinobacillus |
|  |  | 11 | Firmicutes;Bacilli;Bacillales;Staphylococcaceae;Gemella |
|  |  | 50 | Firmicutes;Clostridia;Clostridiales;Lachnospiraceae;Johnsonella |
|  |  | 26 | Bacteroidetes;Bacteroidia;Bacteroidales;Prevotellaceae;Prevotella |
|  |  | 18 | Actinobacteria;Actinobacteria;Actinomycetales;Micrococcaceae;Rothia |
|  |  | 21 | Proteobacteria;Betaproteobacteria;Neisseriales;Neisseriaceae;Neisseria |
|  |  | 39 | Bacteroidetes;Bacteroidia;Bacteroidales;Prevotellaceae;Prevotella |
|  |  | 68 | Firmicutes;Clostridia;Clostridiales;Lachnospiraceae;Catonella |
|  |  | 56 | Fusobacteria;Fusobacteria;Fusobacteriales;Leptotrichiaceae;Leptotrichia |
| Stool | V1-V3 | 39 | Firmicutes;Clostridia;Clostridiales;Lachnospiraceae;Pseudobutyrivibrio |
|  |  | 29 | Firmicutes;Clostridia;Clostridiales;Lachnospiraceae;Coprococcus |
|  |  | 103 | Firmicutes;Clostridia;Clostridiales;Lachnospiraceae;Hespellia |
|  |  | 30 | Firmicutes;Clostridia;Clostridiales;Ruminococcaceae;Faecalibacterium |
|  |  | 94 | Firmicutes;Clostridia;Clostridiales;Ruminococcaceae;Oscillibacter |
|  |  | 17 | Bacteroidetes;Bacteroidia;Bacteroidales;Bacteroidaceae;Bacteroides |
|  |  | 69 | Bacteroidetes;Bacteroidia;Bacteroidales;Bacteroidaceae;Bacteroides |
|  | V3-V5 | 27 | Bacteroidetes;Bacteroidia;Bacteroidales;Bacteroidaceae;Bacteroides |
|  |  | 17 | Bacteroidetes;Bacteroidia;Bacteroidales;Bacteroidaceae;Bacteroides |
|  |  | 42 | Firmicutes;Clostridia;Clostridiales;Ruminococcaceae;Faecalibacterium |
|  |  | 167 | Firmicutes;Clostridia;Clostridiales;Lachnospiraceae;Roseburia |
|  |  | 93 | Firmicutes;Clostridia;Clostridiales;Ruminococcaceae;Subdoligranulum |
| Subgingival plaque | V1-V3 | 2 | Firmicutes;Bacilli;Lactobacillales;Streptococcaceae;Streptococcus |
|  |  | 27 | Fusobacteria;Fusobacteria;Fusobacteriales;Fusobacteriaceae;Fusobacterium |
|  |  | 7 | Firmicutes;Clostridia;Clostridiales;Veillonellaceae;Veillonella |
|  |  | 5 | Firmicutes;Bacilli;Lactobacillales;Streptococcaceae;Streptococcus |
|  |  | 14 | Proteobacteria;Epsilonproteobacteria;Campylobacterales;Campylobacteraceae;Campylobacter |
|  |  | 31 | Bacteroidetes;Flavobacteria;Flavobacteriales;Flavobacteriaceae;Chryseobacterium |
|  |  | 24 | Actinobacteria;Actinobacteria;Actinomycetales;Actinomycetaceae;Actinomyces |
|  |  | 28 | Bacteroidetes;Flavobacteria;Flavobacteriales;Flavobacteriaceae;Capnocytophaga |
|  |  | 10 | Actinobacteria;Actinobacteria;Actinomycetales;Micrococcaceae;Rothia |
|  |  | 37 | Bacteroidetes;Flavobacteria;Flavobacteriales;Flavobacteriaceae;Capnocytophaga |
|  | V3-V5 | 9 | Fusobacteria;Fusobacteria;Fusobacteriales;Fusobacteriaceae;Fusobacterium |
|  |  | 2 | Firmicutes;Bacilli;Lactobacillales;Streptococcaceae;Streptococcus |
|  |  | 23 | Actinobacteria;Actinobacteria;Actinomycetales;Actinomycetaceae;Actinomyces |
|  |  | 4 | Firmicutes;Clostridia;Clostridiales;Veillonellaceae;Veillonella |
|  |  | 21 | Proteobacteria;Betaproteobacteria;Neisseriales;Neisseriaceae;Neisseria |
|  |  | 16 | Proteobacteria;Gammaproteobacteria;Pasteurellales;Pasteurellaceae;Pasteurella |
| Supragingival plaque | V1-V3 | 2 | Firmicutes;Bacilli;Lactobacillales;Streptococcaceae;Streptococcus |
|  |  | 5 | Firmicutes;Bacilli;Lactobacillales;Streptococcaceae;Streptococcus |
|  |  | 7 | Firmicutes;Clostridia;Clostridiales;Veillonellaceae;Veillonella |
|  |  | 27 | Fusobacteria;Fusobacteria;Fusobacteriales;Fusobacteriaceae;Fusobacterium |
|  |  | 31 | Bacteroidetes;Flavobacteria;Flavobacteriales;Flavobacteriaceae;Chryseobacterium |
|  |  | 28 | Bacteroidetes;Flavobacteria;Flavobacteriales;Flavobacteriaceae;Capnocytophaga |
|  |  | 24 | Actinobacteria;Actinobacteria;Actinomycetales;Actinomycetaceae;Actinomyces |
|  |  | 10 | Actinobacteria;Actinobacteria;Actinomycetales;Micrococcaceae;Rothia |
|  |  | 37 | Bacteroidetes;Flavobacteria;Flavobacteriales;Flavobacteriaceae;Capnocytophaga |
|  |  | 19 | Firmicutes;Bacilli;Lactobacillales;Carnobacteriaceae;Granulicatella |
|  |  | 38 | Fusobacteria;Fusobacteria;Fusobacteriales;Leptotrichiaceae;Leptotrichia |
|  |  | 43 | Proteobacteria;Betaproteobacteria;Neisseriales;Neisseriaceae;Kingella |
|  |  | 65 | Actinobacteria;Actinobacteria;Actinomycetales;Corynebacteriaceae;Corynebacterium |
|  | V3-V5 | 2 | Firmicutes;Bacilli;Lactobacillales;Streptococcaceae;Streptococcus |
|  |  | 9 | Fusobacteria;Fusobacteria;Fusobacteriales;Fusobacteriaceae;Fusobacterium |
|  |  | 21 | Proteobacteria;Betaproteobacteria;Neisseriales;Neisseriaceae;Neisseria |
|  |  | 4 | Firmicutes;Clostridia;Clostridiales;Veillonellaceae;Veillonella |
|  |  | 23 | Actinobacteria;Actinobacteria;Actinomycetales;Actinomycetaceae;Actinomyces |
|  |  | 113 | Bacteroidetes;Flavobacteria;Flavobacteriales;Flavobacteriaceae;Cloacibacterium |
|  |  | 13 | Firmicutes;Bacilli;Lactobacillales;Carnobacteriaceae;Granulicatella |
|  |  | 16 | Proteobacteria;Gammaproteobacteria;Pasteurellales;Pasteurellaceae;Pasteurella |
|  |  | 18 | Actinobacteria;Actinobacteria;Actinomycetales;Micrococcaceae;Rothia |
|  |  | 36 | Bacteroidetes;Flavobacteria;Flavobacteriales;Flavobacteriaceae;Capnocytophaga |
|  |  | 6 | Firmicutes;Bacilli;Lactobacillales;Streptococcaceae;Streptococcus |
|  |  | 24 | Bacteroidetes;Flavobacteria;Flavobacteriales;Flavobacteriaceae;Capnocytophaga |
|  |  | 19 | Proteobacteria;Gammaproteobacteria;Pasteurellales;Pasteurellaceae;Actinobacillus |
|  |  | 32 | Actinobacteria;Actinobacteria;Actinomycetales;Corynebacteriaceae;Corynebacterium |
|  |  | 15 | Actinobacteria;Actinobacteria;Actinomycetales;Corynebacteriaceae;Corynebacterium |
| Throat | V1-V3 | 2 | Firmicutes;Bacilli;Lactobacillales;Streptococcaceae;Streptococcus |
|  |  | 5 | Firmicutes;Bacilli;Lactobacillales;Streptococcaceae;Streptococcus |
|  |  | 7 | Firmicutes;Clostridia;Clostridiales;Veillonellaceae;Veillonella |
|  |  | 27 | Fusobacteria;Fusobacteria;Fusobacteriales;Fusobacteriaceae;Fusobacterium |
|  |  | 29 | Firmicutes;Clostridia;Clostridiales;Lachnospiraceae;Oribacterium |
|  |  | 14 | Proteobacteria;Epsilonproteobacteria;Campylobacterales;Campylobacteraceae;Campylobacter |
|  |  | 12 | Proteobacteria;Betaproteobacteria;Neisseriales;Neisseriaceae;Neisseria |
|  |  | 34 | Actinobacteria;Actinobacteria;Actinomycetales;Actinomycetaceae;Actinomyces |
|  |  | 38 | Fusobacteria;Fusobacteria;Fusobacteriales;Leptotrichiaceae;Leptotrichia |
|  |  | 16 | Bacteroidetes;Bacteroidia;Bacteroidales;Prevotellaceae;Prevotella |
|  |  | 19 | Firmicutes;Bacilli;Lactobacillales;Carnobacteriaceae;Granulicatella |
|  |  | 8 | Firmicutes;Bacilli;Bacillales;Staphylococcaceae;Gemella |
|  |  | 78 | Actinobacteria;Actinobacteria;Actinomycetales;Actinomycetaceae;Actinomyces |
|  |  | 41 | Bacteroidetes;Bacteroidia;Bacteroidales;Prevotellaceae;Prevotella |
|  |  | 13 | Actinobacteria;Actinobacteria;Actinomycetales;Micrococcaceae;Rothia |
|  | V3-V5 | 2 | Firmicutes;Bacilli;Lactobacillales;Streptococcaceae;Streptococcus |
|  |  | 4 | Firmicutes;Clostridia;Clostridiales;Veillonellaceae;Veillonella |
|  |  | 9 | Fusobacteria;Fusobacteria;Fusobacteriales;Fusobacteriaceae;Fusobacterium |
|  |  | 6 | Firmicutes;Bacilli;Lactobacillales;Streptococcaceae;Streptococcus |
|  |  | 10 | Bacteroidetes;Bacteroidia;Bacteroidales;Prevotellaceae;Prevotella |
|  |  | 16 | Proteobacteria;Gammaproteobacteria;Pasteurellales;Pasteurellaceae;Pasteurella |
|  |  | 13 | Firmicutes;Bacilli;Lactobacillales;Carnobacteriaceae;Granulicatella |
|  |  | 14 | Actinobacteria;Actinobacteria;Actinomycetales;Actinomycetaceae;Actinomyces |
|  |  | 11 | Firmicutes;Bacilli;Bacillales;Staphylococcaceae;Gemella |
|  |  | 8 | Proteobacteria;Betaproteobacteria;Neisseriales;Neisseriaceae;Neisseria |
|  |  | 50 | Firmicutes;Clostridia;Clostridiales;Lachnospiraceae;Johnsonella |
|  |  | 33 | Firmicutes;Clostridia;Clostridiales;Lachnospiraceae;Oribacterium |
|  |  | 25 | Bacteroidetes;Bacteroidia;Bacteroidales;Bacteroidaceae;Bacteroides |
| Tongue dorsum | V1-V3 | 5 | Firmicutes;Bacilli;Lactobacillales;Streptococcaceae;Streptococcus |
|  |  | 2 | Firmicutes;Bacilli;Lactobacillales;Streptococcaceae;Streptococcus |
|  |  | 7 | Firmicutes;Clostridia;Clostridiales;Veillonellaceae;Veillonella |
|  |  | 29 | Firmicutes;Clostridia;Clostridiales;Lachnospiraceae;Oribacterium |
|  |  | 14 | Proteobacteria;Epsilonproteobacteria;Campylobacterales;Campylobacteraceae;Campylobacter |
|  |  | 19 | Firmicutes;Bacilli;Lactobacillales;Carnobacteriaceae;Granulicatella |
|  |  | 16 | Bacteroidetes;Bacteroidia;Bacteroidales;Prevotellaceae;Prevotella |
|  |  | 27 | Fusobacteria;Fusobacteria;Fusobacteriales;Fusobacteriaceae;Fusobacterium |
|  |  | 8 | Firmicutes;Bacilli;Bacillales;Staphylococcaceae;Gemella |
|  |  | 34 | Actinobacteria;Actinobacteria;Actinomycetales;Actinomycetaceae;Actinomyces |
|  |  | 13 | Actinobacteria;Actinobacteria;Actinomycetales;Micrococcaceae;Rothia |
|  |  | 78 | Actinobacteria;Actinobacteria;Actinomycetales;Actinomycetaceae;Actinomyces |
|  |  | 38 | Fusobacteria;Fusobacteria;Fusobacteriales;Leptotrichiaceae;Leptotrichia |
|  |  | 120 | Actinobacteria;Actinobacteria;Actinomycetales;Actinomycetaceae;Actinomyces |
|  |  | 12 | Proteobacteria;Betaproteobacteria;Neisseriales;Neisseriaceae;Neisseria |
|  | V3-V5 | 6 | Firmicutes;Bacilli;Lactobacillales;Streptococcaceae;Streptococcus |
|  |  | 4 | Firmicutes;Clostridia;Clostridiales;Veillonellaceae;Veillonella |
|  |  | 10 | Bacteroidetes;Bacteroidia;Bacteroidales;Prevotellaceae;Prevotella |
|  |  | 9 | Fusobacteria;Fusobacteria;Fusobacteriales;Fusobacteriaceae;Fusobacterium |
|  |  | 2 | Firmicutes;Bacilli;Lactobacillales;Streptococcaceae;Streptococcus |
|  |  | 16 | Proteobacteria;Gammaproteobacteria;Pasteurellales;Pasteurellaceae;Pasteurella |
|  |  | 14 | Actinobacteria;Actinobacteria;Actinomycetales;Actinomycetaceae;Actinomyces |
|  |  | 13 | Firmicutes;Bacilli;Lactobacillales;Carnobacteriaceae;Granulicatella |
|  |  | 25 | Bacteroidetes;Bacteroidia;Bacteroidales;Bacteroidaceae;Bacteroides |
|  |  | 33 | Firmicutes;Clostridia;Clostridiales;Lachnospiraceae;Oribacterium |
|  |  | 11 | Firmicutes;Bacilli;Bacillales;Staphylococcaceae;Gemella |
|  |  | 50 | Firmicutes;Clostridia;Clostridiales;Lachnospiraceae;Johnsonella |
|  |  | 8 | Proteobacteria;Betaproteobacteria;Neisseriales;Neisseriaceae;Neisseria |
| Vaginal introitus | V1-V3 | N/A |  |
|  | V3-V5 | 6 | Firmicutes;Bacilli;Lactobacillales;Streptococcaceae;Streptococcus |
