# Supplementary material for: A Core Human Microbiome as Viewed through 16S rRNA Sequence Clusters
Source: PLoS One. 2012 Jun 13;7(6):e34242. doi: 10.1371/journal.pone.0034242 (PMC3374614; doi:10.1371/journal.pone.0034242)
Supplement: Table S4 — Example species-level taxonomy for selected OTUs reported in the section Patterns in the healthy human microbiome within genera . Sequence tags were initially identified with RDP only to genus or higher, and then each OTU was assigned a consensus of all taxa present. These example species-level taxa assignments are based on BLAST to nt, excluding uncultured and environmental sample sequences for the three most abundant sequences in each OTU. All taxa from sequences matching the best query coverage and percent identity were considered most likely species assignments. (DOCX) [file pone.0034242.s005.docx]

Table S4

| **Taxonomy** | **OTU #** | **Taxa of top BLAST Hits** | **BLAST Query Coverage / Percent Identity** |
| --- | --- | --- | --- |
| Actinomycetales | V1-V3 #35 | *Streptomyces aini, S. axinellae, S. chugwhensis, S. endus, S. paucisporeus, Streptacidiphilus sp., Actinomyces* | (98-99)/(97-98) |
| Actinomycetales | V1-V3 #65 | *Corynebacterium durum* | 100(98-100) |
| Actinomycetales | V1-V3 #96 | *Actinomyces gravenitzii* | 100/100 |
| Actinomycetales | V1-V3 #151 | *Actinomyces sp.* | 100(95-100) |
| Actinomycetales | V1-V3 #165 | *multiple* | 100/100 |
| Actinomycetales | V1-V3 #209 | *Corynebacterium kroppenstedtii* | 100/99 |
| Actinomycetales | V1-V3 #308 | *Mycobacterium abscessus, M. bolletii, M. chelonaev(ATCC), M. franklinii, M. fuerth, M. massiliense* | 100/100 |
| Bacteroides | V3-V5 #17 | *B. dorei, B.vulgatus* | 100/100 |
| Bacteroides | V3-V5 #45 | *B. stercoris (ATCC)* | 100/99 |
| Bacteroides | V3-V5 707 | *B.dorei, B.vulgatus (ATCC)* | (94-100)/100 |
| Bacteroides | V3-V5 #1004 | *B.acidifaciens, B. facecis, B. fragilis (ATCC), B. ovatus, B. salyersiae, B. thetaiotamicron (ATCC)* | 100/98 |
| Clostridium | V3-V5 #72 | *C. acetobutylicum, beijerinckii, butyricum, diolis, roseum* | 100/100 |
| Clostridium | V3-V5 #184 | *perfringens(ATCC)* | 100/99 |
| Corynebacterium | V3-V5 #12 | *C. accolens (ATCC), C. fastidium, C. pseudogenitalium, C. segmentosu, C. tuberculostearicum* | 100/100 |
| Corynebacterium | V3-V5 #15 | *C. matruchotii* | 100/100 |
| Corynebacterium | V3-V5 #101 | *C. afermentans, C. coyleae, C. imitans, C. mucifaciens, C. ureicelerivorans* | 100/100 |
| Corynebacterium | V3-V5 #188 | *C. argentoratense* | 100/100 |
| Corynebacterium | V3-V5 #418 | *C. glucuronolyticum* | 100/100 |
| Fusobacterium | V3-V5 #9 | *F. periodonticum (ATCC)* | 100/100 |
| Fusobacterium | V3-V5 #523 | *F. canifelinum, F. fusiforme (ATCC), F. nucleatum, F. naviforme, F. periodonticum, F. simiae, Filifactor alocis (ATCC)* | 98/99 |
| Fusobacterium | V3-V5 #738 | *F. canifelinum, F. naviforme, F. nucleatum (ATCC), F. periodonticum (ATCC), F. simiae, Filifactor alocis (ATCC)* | 96/99, 95/100 |
| Fusobacterium | V3-V5 #1314 | *F. equinum, F. necrophorum (ATCC)* | 100/100 |
| Neisseria | V3-V5 #8 | *N. favescens, N. meningitidis, N. mucosa, N. perflava, N. subflava* | 100/100 |
| Neisseria | V3-V5 #21 | *N.flava, N. mucosa, N. sicca, Morococcus cerebrosus* | 100/100 |
| Neisseria | V3-V5 #98 | *Neisseria sp.* | 100/100 |
| Neisseria | V3-V5 #220 | *N. bacilliformis (ATCC)* | 100/100 |
| Neisseriaceae | V3-V5 #40 | *Kingella kingae (ATCC), N. flavescens, N. meningitidis, N. mucosa, N. perflava, N. subflava, Simonsiella muelleri (ATCC)* | (98-100)/93 |
| Neisseriaceae | V3-V5 #85 | *Kingella kingae (ATCC), N. flavescens, N. meningitidis, N. mucosa, N. perflava, N. subflava, Simonsiella muelleri (ATCC)* | (98-100)/(91-93) |
| Neisseriaceae | V3-V5 #843 | *Kingella kingae (ATCC), K. oralis, N. animalis, N. weaveri, N. zoodegmatis, Simonsiella crassa (ATCC)* | 97/99 |
| Neisseriaceae | V3-V5 #918 | *Alysiella filiformis, N. canis, N. wordsworthii, Simonsiella crassa (ATCC)* | 100/98 |
| Neisseriaceae | V3-V5 #1001 | *Kingella dentrificans (ATCC), Neisseria. zoodegmatis, Simonsiella crassa (ATCC)* | 100/98 |
| Pasteurellaceae | V3-V5 #16 | *Haemophilus parainfluenzae* | 100/100 |
| Pasteurellaceae | V3-V5 #19 | *Haemophilus haemolyticus* | 100/100 |
| Pasteurellaceae | V3-V5 #1185 | *N. flavescens, N. meningitidis, N. mucosa, N. perflava, N. pharyngis, N. polysaccharea, N. subflava, etc.* | 100/(92-93) |
| Pasteurellaceae | V3-V5 #1511 | *Actinobacillus, Aggregatibacter, Haemophilus, Mannheimia, Nicoletella, Pasteurella* | 97/(99-100) |
| Pasteurellaceae | V3-V5 #1725 | *Streptococcus infantis, S. mitis, S. oralis (ATCC), S. pneumoniae (ATCC), S. pseudopneumoniae, S. oligofermentans* | 100/98 |
| Prevotella | V3-V5 #10 | *P. melaninogenica (ATCC)* | 100/100 |
| Prevotella | V3-V5 #26 | *P. pallens* | 100/100 |
| Prevotella | V3-V5 #67 | *P. nanceinensis* | 100/100 |
| Prevotellaceae | V3-V5 #34 | *Prevotella sp.* | 100/100 |
| Prevotellaceae | V3-V5 #149 | *Prevotella sp.* | 100/100 |
| Prevotellaceae | V3-V5 #195 | *Prevotella sp.* | 100/98 |
| Prevotellaceae | V3-V5 #214 | *Prevotellaceae* | 100/91 |
| Prevotellaceae | V3-V5 #241 | *Prevotella sp.* | 100/99 |
| Prevotellaceae | V3-V5 #333 | *P. histolica, P. melaninogenica (ATCC), P. scopos, P. veroralis* | 100/95 |
| Prevotellaceae | V3-V5 #457 | *Prevotella sp.* | 100/(91-92) |
| Streptococcus | V3-V5 #2 | *S. pneumoniae, mitis (ATCC), S. infantis, S. oralis (ATCC), sanguinis (ATCC)* | 100 / 100 |
| Streptococcus | V3-V5 #6 | *S. salivarius (ATCC), S. thermophilus (ATCC), S. vestibularis (ATCC)* | 100/100 |
| Streptococcus | V3-V5 #60 | *S. mutans* | 100/100 |
| Streptococcus | V3-V5 #596 | *S. pseudopneumoniae, S. pneumoniae, S. infantis, S. mitis, S. oralis* | 100/(98-100) |
